# Investigation of composed charged particles with suspension of ternary hybrid nanoparticles in 3D-power law model computed by Galerkin algorithm

**DOI:** 10.1038/s41598-023-41449-y

**Published:** 2023-09-12

**Authors:** Umar Nazir, Kanit Mukdasai, Muhammad Sohail, Abha Singh, Mohammed Theeb Alosaimi, Mashael Alanazi, Ayele Tulu

**Affiliations:** 1 Department of Mathematics, Faculty of Science, Khon Kaen University, Khon Kaen, 40002 Thailand; 2 Institute of Mathematics, Khwaja Fareed University of Engineering & Information Technology, Rahim Yar Khan, 64200 Pakistan; 3 Department of Basic Sciences, College of Sciences and Theoretical Studies, Dammam-branch, Saudi Electronic University, Riyadh, Saudi Arabia; 4 College of science and health professions, King Saud Bin Abdulaziz University for health Sciences, Riyadh, Saudi Arabia; 5 King Abdullah International Medical Research (KAIMRC), Riyadh, Saudi Arabia; 6 Ministry of National Guard-Health Affairs (MNGHA), Riyadh, Saudi Arabia; 7 Department of Mathematics, CNCS, Ambo University, Ambo, Ethiopia

**Keywords:** Mathematics and computing, Nanoscience and technology

## Abstract

Transport of heat visualizes a vital role in many industrial developments. Current study is discussing the role of Joule heating, solar thermal radiation, heat generation/absorption, reactions (homogeneous and heterogeneous) with variable thermal conductivity on partially ionized power law material past over a three-dimensional heated stretched surface. The power law model is assumed to have the thermal characteristics of ethylene glycol material. The phenomenon of momentum and energy balance is derived in Cartesian coordinates and developed PD (partial differential)-equations. Swimming pools, solar collectors, food processing, electronic gadgets, cooling systems, magnetic field measurement, computer chips, thermal enhancement, semiconductor characterization, nuclear fusion research and other physical applications are examples of ongoing research. The principle of boundary layer simplified the governing problem. The complex coupled PD (partial differential)-equations have been converted into ordinary differential equations OD (ordinary differential)-equations by using appropriate similarity transformation. The converted boundary value problem is complex and highly nonlinear which does not have the exact solution. The approximate solution is computed numerically via finite element scheme (FES) which is coded in MAPLE 18.0 symbolic package. The convergence of the scheme is established through grid independent survey and the solution is plotted against numerous involved parameters. Thermal performance produced by $Si{O}_{2}$-$Ti{O}_{2}$-${Al}_{2}{O}_{3}$/EG is higher thermal performance produced by $Si{O}_{2}$-$Ti{O}_{2}$/EG. Ion slip and Hall forces are responsible for generating Joule heating mechanism that is responsible for reduction of velocity curve and generating shear stresses. Hence, tangential stresses are declined against increasing ${\beta }_{i}$ and ${\beta }_{e}.$

## Introduction

Inclusion of nanoparticles boosts thermal performance and it has numerous applications in different systems. Researchers have done extensive work on flow with heat transfer. For instance, Nadeem et al.^1^ examined the flow demeanor of the Casson fluid towards a penetrable linear stretched sheet. Haq et al.^2^ considered the slip effects along with thermal radiation in the Magneto-hydrodynamic stagnation point Nano fluidic flow behavior subjected to a stretched sheet. Boundary layer Nano-fluidic demeanor configured by moveable surface was addressed by Bachok et al.^3^. Moreover, Ahmed and Pop^4^ also considered BL Nano-fluidic flow and investigated the porous enclosure. Rasool et al.^5^ used the Powell-Eyring Nano fluidic flow model under the salient distinctive features of chemical reaction. Shafiq et al.^6^ modeled the 3D Darcy Forchheimer Nano- fluidic flow subjected to a rotatory system exposed to the convective limiting conditions along with impacts of thermal slip. Haq et al.^7^ modeled the convective transport of heat analysis in the magnetic slip flow demeanor containing Carbon Nanotubes. Oudina et al.^8^ considered the thermal source of having distinctive lengths for the numerical modeling based on the hydrodynamic stability in association with cylindrical annular. Furthermore, Nour et al.^9^ used the porosity medium and proposed the numerical approach for entropy production as well as MHD convection towards the hybrid Nano fluidic flow behavior. Marzougui et al.^10^ also studied the entropy production analysis in the MHD Copper- water based Nano-fluidic flow towards a cavity associated with chamfers. Tassaddiq et al.^11^ modeled the mass as well as heat transportation in the hybrid Nano fluidic flow subjected to a stretched surface. Ramesh et al.^12^ work based on the Kinetic analysis of Co oxidation with reduction (TPR) experiments on distinctive Manganese oxides (MnO_x_). In view of Titania Nano fluids within the distinctive base fluids associated with cylindrical annulus exposed to the diverse heat source was studied by Oudina et al.^13^. Chu et al.^14^ captured the role of Cattaneo-Christove dual diffusive theory along with a radiative heat flow model subjected to a stretched surface. Shah et al.^15^ estimated the heat transportation in the fluid flow of second grade subjected to Caputo- Fabrizio fractional derivative technique along the unbounded oscillatory vertical plate. Khan et al.^16^ analyzed and presented the thermal radiation characteristics in the Nano fluidic flow demeanor in a thick moveable penetrating stretched or shrinking surface under the convex as well as concave effects. Hajizadeh et al.^17^ considered the free convective Nano fluidic flow associated with binary vertical plates subjected to damped thermal flux and radiations. Moreover, Shah et al.^18^ examined the role of thermal radiations amongst the dual parallel plates subjected to a rotatory system. Shah et al.^19^ work based on the modeling of heat as well as mass transportation based on the conductor non-Newtonian flow demeanor of third grade amongst the binary parallel under the impacts of Brownian motion and thermophoresis phenomenon. Abdelmalek et al.^20^ used magnetized Williamson Nano-fluidic flow to model configured by a stretching cylinder under the affecting features of activation energy, second order slip impacts along with thermal conductivity in his investigation. Alwatban et al.^21^ study based on the exponentially moveable surface to investigate the rheological results in the Eyring Powell Nano fluidic flow demeanor under the consideration of thermal radiation along with activation energy impacts. Whereas, Ahmed at al.^22^ analyzed the transient convection fractional Nano fluidic flow demeanor amongst the parallel plates. Khan et al.^23^ modeled the heat transportation and time reliant stagnation point flow (SPF) in the flow of hybrid nanofluid flow configured by a penetrable shrinking/stretched surface.

The study of fluid flow within the slip flow scheme is considered to be extremely significant in multitudinous engineering fields namely magneto-hydrodynamic power generators, accelerators, refrigeration coils and electric transformers. In numerous cases, the Hall Effect within the ion-slip current is considered to be of fragile role in fluid study. In general, Hall current influence depicts an energetic disposition as a reason Hall current is extremely high. Hall parameter is defined as the relationship of rate of electron cyclotron within the collision of rate of atom electrons. Whereas, ion slip is termed as combined effects of ion as well as electrons flow velocities. For modelling heat as well as mass transmission, analysis in the Nano fluidic flow demeanor of Carreau-Yasuda Hybrid fluid in view of combined hall and ion slip impacts was reported by Rana et al.^24^. To model the completely developed magneto-hydrodynamic laminar fluid flow immersed in a channel under the Hall current and ion slip influence was proposed by Javeri et al.^25^. Under the consideration of Hall along with ion slip impacts were observed and analyzed by Soundalgekar et al.^26^ towards the completely developed flow. Moreover, the 3D heat transportation phenomenon was modeled by Nawaz et al.^27^ in the combination of nanoparticles along with magneto-hydrodynamic micro polar plasma subjected to Hall effects and ion slip current. Asghar et al.^28^ considered the peristaltic transfer regarding to MHD fluid flow on account of symmetric as well as asymmetric channel was analyzed under the Hall and ion slip influence. However, they also modeled the heat transmission phenomenon under the consideration of Hall current and ion slip impacts. Several other contributions on transport phenomena are covered in^29–32^ and the studies mentioned there. Shahzad et al.^33^ experienced thermal growth using three kinds of nanostructures and viscous dissipation on heated disk with thermal radiation. They adopted a numerical approach named as Keller box scheme for numerical consequences. Shamshuddin et al.^34^ reported energy transition in Newtonian fluid adding ternary hybrid nanoparticles with charged particles and base fluid (polymer). They assumed several aspects of viscous dissipation and thermal radiation utilized analytically. Ferdows et al.^35^ estimated thermal features and cooling performance in Newtonian fluid with mix conditions towards surface adding nanofluids. They found that thermal conductivity can be improved by adding silver nanofluid. Salawu et al.^36^ developed a 2-dimensional model regarding Eyring Powell liquid utilizing over surface solar thermal radiation and nanofluid. It was found that the temperature curve is increased by enhancing values of Eckert number and solar thermal radiation number. Rahman et al.^37^ reported numerical simulations of hybrid nanoparticles using Eckert number and viscous dissipation. They considered aluminum oxide, water and copper oxide towards a plate simulated numerically using a shooting approach. Mohapatra et al.^38^ discovered thermal features of unsteady flow of micropolar fluid across an inclined plate in the presence of dissipative heat energy via FEA (finite element approach). Sheri et al.^39^ adopted FEM on magnetohydrodynamic flow micropolar fluid in the presence of Hall current towards the vertical plate. They concluded thickness regarding momentum boundary is enhanced by increasing Eckert number and Hall parameter. Shamshuddin et al.^40^ concentrated on creating a mathematical model and researching the rheological characteristics of a Casson-type nanofluid flow that was interacting chemically. They discovered that lower values of the Casson fluid parameter led to a drop in fluid temperature and velocity. Usman et al.^41^ investigated the interactions between the gyrotactic swimming abilities of the microorganisms and the rheological impacts of the Eyring Powell nanofluid. This study focused on the bioconvection fluid flow phenomena that happens across the surface of a Riga plate encased in a porous material. Khan et al.^42^ evaluated bioconvection in Maxwell nanofluids contained by a stretched surface, they assumed especially studied theoretical research that emphasizes the impact of partial slip and temperature-dependent viscosity. The goal of the study is to better understand these variables' effects on the bioconvection process and overall heat transfer characteristics in the nanofluid system by taking them into account. Alsallami et al.^43^ optimized entropy generation and flow mechanism considering heating processes in second-grade considering over a disk. Lin et al.^44^ discussed power-law ferrofluid over a rotatory stretchable surface of steady three-dimensional boundary layer flow close to the stagnation point and heat transmission. They analyzed Lorentz force affects the flow behavior as well as how nonlinear thermal radiation affects the temperature distribution. The works based on heat transfer are studied in^45–50^.

Prime objective of the present model is to characterize thermal features of Power law martial with charged particles (ion slip and Hall currents) under Darcy’s Forchhiermer theory, heterogeneous and homogeneous reactions across a 3D vertical plate. Development of the problem fills following gaps.

- 3D vertical plate experiences titanium dioxide, aluminum oxide and silicon dioxide in ethylene glycol along with charged particles in Power law material adding variable thermal characteristics using slip conditions are discussed;
- Heterogeneous reaction, heat source, homogeneous reaction and solar thermal radiations are analyzed;
- Joule heating, Darcy’s Forchhiermer magnetic field are addressed. It is revised that according to literature review, such a complex model is not investigated yet implementing a finite element scheme as compared to works^51,52^.

**Figure 1:**
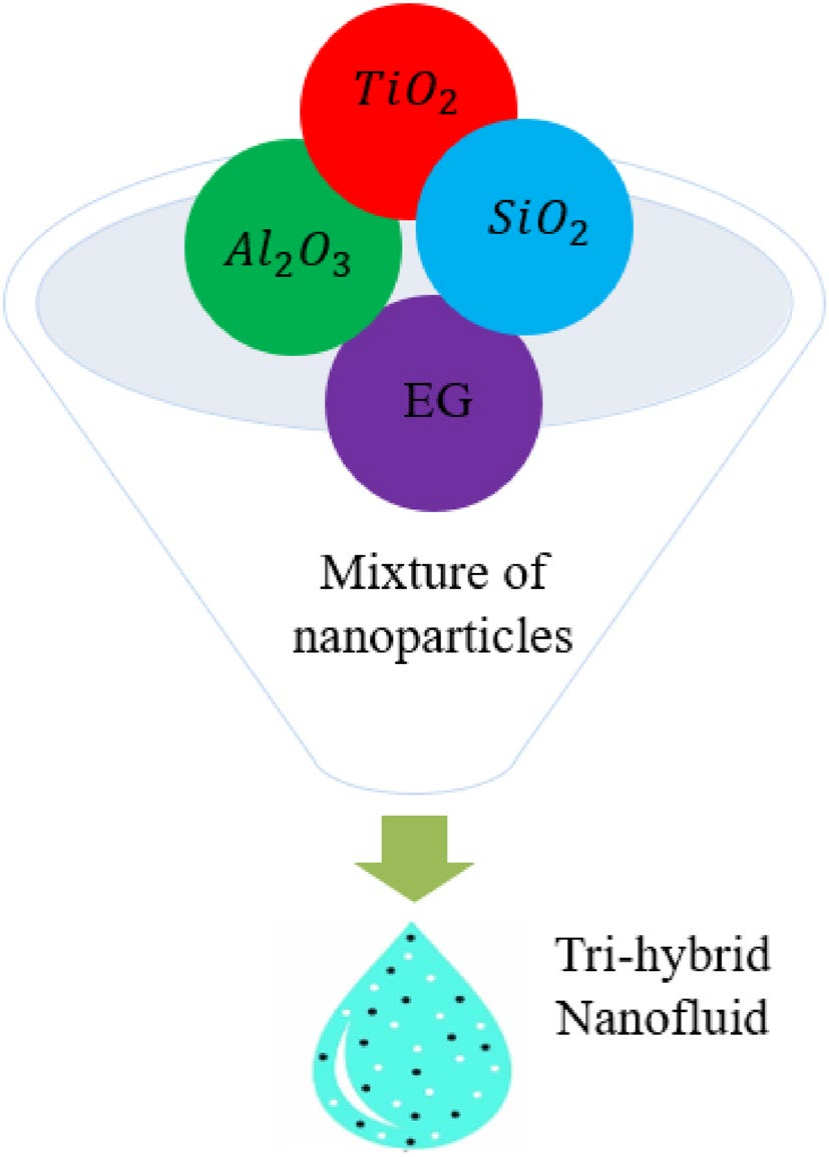
Diagram of tri-hybrid nanoparticles.

Existing studies reveals that no work has been done by considering partially ionize theory for Power law model over a bidirectional stretching sheet by using nanoparticles. This attempt fills this gap. Comprehensive literature is listed in section one, section covers the modelling and finite element analysis is explained in section three and results are analyzed in section four. Figure 1 demonstrates mixture of different nanoparticles with base fluid.

## Modeling and mathematical procedure

**Figure 2:**
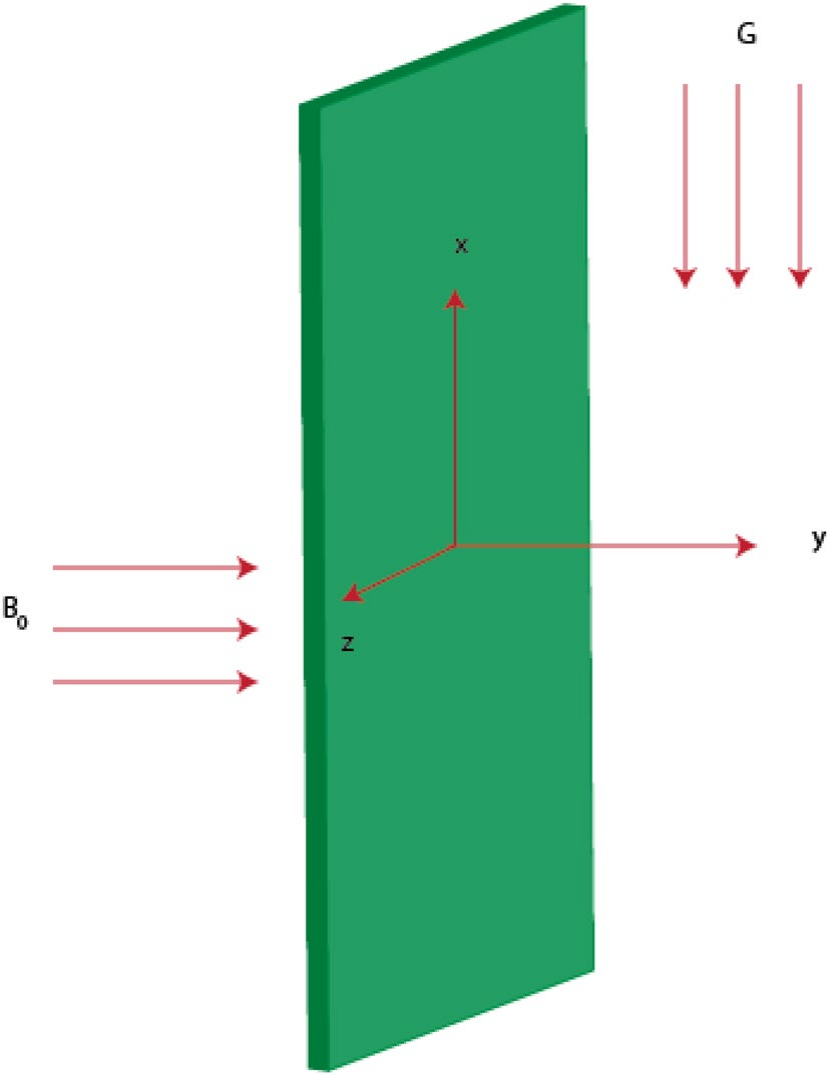
3D stretching surface of problem.

Features in 3D a power law model over a heated surface are analyzed involving reactions based on heterogenous and homogeneous. The forces related to ion slip and Hall are considered into fluidic particles. Thermal energy takes place involving Joule heating phenomena, solar thermal radiation and heat source. The suspension of three type’s nanomaterial in ethylene glycol (base fluid) is inserted into fluidic particles. Moreover, induced phenomena into fluidic particles are produced using movement of walls in both directions. Thermal conductivity in terms of variables is inserted in the presence of tri-hybridized nanoparticles as shown in Fig. 2. Thermal correlations regarding thermal properties in base fluid are tabulated by Fig. 1. BLS are utilized in basic laws whereas formulated PDEs^51,52^ are

1 $$\frac{\partial V}{\partial y}+\frac{\partial U}{\partial x}+\frac{\partial W}{\partial z}=0,$$

2 $$U\frac{\partial U}{\partial x}+V\frac{\partial U}{\partial y}+W\frac{\partial U}{\partial z}=\frac{{K}_{1}}{{\rho }_{thnf}}\frac{\partial }{\partial Z}\left({\left|\frac{\partial U}{\partial z}\right|}^{m-1}\frac{\partial U}{\partial z}\right)+\frac{G}{{\rho }_{Thnf}}{\left(\beta \rho \right)}_{Thnf}\left({T}_{\infty }-T\right) +\frac{{\sigma }_{Thnf}{\left({B}_{0}\right)}^{2}}{{\rho }_{Thnf}\left[{\left({\beta }_{e}\right)}^{2}+{\left(1+{\beta }_{e}{\beta }_{i}\right)}^{2}\right]}\left[{\beta }_{e}V-\left(1+{\beta }_{e}{\beta }_{i}\right)U\right]-\frac{{F}_{st}}{{\left({k}^{*}\right)}^\frac{1}{2}}{U}^{2}-\frac{{\nu }_{Thnf}}{{k}^{*}}{F}_{st}U,$$

3 $$U\frac{\partial V}{\partial x}+V\frac{\partial V}{\partial y}+W\frac{\partial V}{\partial z}=\frac{{K}_{1}}{{\rho }_{thnf}}\frac{\partial }{\partial Z}\left({\left|\frac{\partial V}{\partial z}\right|}^{m-1}\frac{\partial V}{\partial z}\right)+\frac{G}{{\rho }_{Thnf}}{\left(\beta \rho \right)}_{Thnf}\left({T}_{\infty }-T\right)-\frac{{\sigma }_{Thnf}{\left({B}_{0}\right)}^{2}}{{\rho }_{Thnf}\left[{\left({\beta }_{e}\right)}^{2}+{\left(1+{\beta }_{e}{\beta }_{i}\right)}^{2}\right]}\left[{\beta }_{e}U-\left(1+{\beta }_{e}{\beta }_{i}\right)V\right]-\frac{{F}_{st}}{{\left({k}^{*}\right)}^\frac{1}{2}}{V}^{2}-\frac{{\nu }_{Thnf}}{{k}^{*}}{F}_{st}V,$$

4 $$V\frac{\partial T}{\partial y}+U\frac{\partial T}{\partial x}+W\frac{\partial T}{\partial z}=\frac{1}{{(\rho {C}_{p})}_{hnf}}\frac{\partial }{\partial z}\left({k}_{hnf}\left(T\right)\frac{\partial T}{\partial z}\right)+\frac{{\sigma }_{Thnf}{\left({B}_{0}\right)}^{2}}{{\rho }_{Thnf}\left[{\left({\beta }_{e}\right)}^{2}+{\left(1+{\beta }_{e}{\beta }_{i}\right)}^{2}\right]}\left[{U}^{2}+{V}^{2}\right]-\frac{{Q}_{0}}{{\left(\rho {C}_{p}\right)}_{Thnf}}\left(T-{T}_{\infty }\right)+\frac{{A}_{sr}}{{\left(\rho {C}_{p}\right)}_{hnf}}exp\left({A}_{sr}z\right),$$

$$U\frac{\partial A}{\partial x}+V\frac{\partial A}{\partial y}+w\frac{\partial A}{\partial z}={D}_{a}\frac{{\partial }^{2}A}{\partial {z}^{2}}-{k}_{c}A{B}^{2},$$

$$U\frac{\partial B}{\partial x}+v\frac{\partial B}{\partial y}+w\frac{\partial B}{\partial z}={D}_{b}\frac{{\partial }^{2}B}{\partial {z}^{2}}+{k}_{c}A{B}^{2},$$

The required boundary conditions are mentiioned^51,52^ as

5 $$U=c\left(x+y\right), V=c\left(x+y\right)\mathrm{ at } z=0, {D}_{a}\frac{\partial A}{\partial z}={k}_{s}A, {D}_{b}\frac{\partial B}{\partial z}={k}_{s}A,$$

$$-{k}_{thnf}\frac{\partial T}{\partial z}=\left({T}_{f}-T\right){h}_{f}, A\to {a}_{0}$$, $$B\to 0,V\to 0,T\to {T}_{\infty }, U\to 0,$$ when $$z\to \infty .$$

Variables transformations^51^ are

$$\theta \left({T}_{f}-{T}_{\infty }\right)=\left(T-{T}_{\infty }\right), U=a\left(x+y\right){F}{\prime}, V=b\left(x+y\right){g}{\prime}, B={a}_{0}H\left(\eta \right),$$

$$A={a}_{0}R\left(\eta \right), \eta ={\left(\frac{b{a}^{n-2}}{{\rho }_{f}}\right)}^{\frac{1}{n+1}}z{x}^{\frac{1-n}{1+n}},w=-a{\left(\frac{b{a}^{n-2}}{{\rho }_{f}}\right)}^{\frac{1}{n+1}}\left[\frac{2n}{n+1}F+\frac{1-n}{1+n}\eta {F}{\prime}+g\right]{x}^{\frac{1-n}{1+n}}.$$

Thermal conductivity under occurrence of hybrid nanofluid is defined as

6 $${K}_{hnf}\left(T\right)={K}_{hnf}\left[1+{\epsilon }_{1}\left(\frac{T-{T}_{\infty }}{{T}_{w}-{T}_{\infty }}\right)\right].$$

Thermal correlations (for ternary hybrid nanomaterials)^29^ are and their values ae listed in Table 1

**Table 1:** Different thermal chrecterstics of EG, $Ti{O}_{2}, Si{O}_{2}$ and ${Al}_{2}{O}_{3}$^29^.

Egine oil	Silicon dioxide	Titanium dioxide	Aluminium oxide
$K\left(0.144\right)$	$K\left(1.4013\right)$	$K\left(8.953\right)$	$K\left(32.9\right)$
$\sigma \left(0.125\times {10}^{-11}\right)$	$\sigma \left(3.5\times {10}^{6}\right)$	$\sigma \left(2.4\times {10}^{6}\right)$	$\sigma \left(5.96\times {10}^{7}\right)$
$\rho \left(884\right)$	$\rho \left(2270\right)$	$\rho \left(4250\right)$	$\rho \left(6310\right)$

7 $${\rho }_{Thnf}=\left(1-{phi}_{1}\right)\left\{\left(1-{phi}_{2}\right)\left[\left(1-{phi}_{3}\right){\rho }_{f}+{phi}_{3}{\rho }_{3}\right]+{phi}_{2}{\rho }_{2}\right\}+{phi}_{1}{\rho }_{1,}$$

8 $$\frac{{\mu }_{f}}{{\left(1-{phi}_{3}\right)}^{2.5}{\left(1-{phi}_{2}\right)}^{2.5}{\left(1-{phi}_{1}\right)}^{2.5}}, \frac{{K}_{hnf}}{{K}_{nf}}=\frac{{K}_{2}+2{K}_{nf}-2{phi}_{1}\left({K}_{nf}-{K}_{2}\right)}{{K}_{2}+2{K}_{nf}+{phi}_{2}\left({K}_{nf}-{K}_{2}\right)},$$

9 $$\frac{{K}_{Thnf}}{{K}_{hnf}}=\frac{{K}_{1}+2{K}_{hnf}-2{phi}_{1}\left({K}_{hnf}-{K}_{1}\right)}{{K}_{1}+2{K}_{hnf}+{phi}_{1}\left({K}_{hnf}-{K}_{1}\right)}, \frac{{K}_{nf}}{{K}_{f}}=\frac{{K}_{3}+2{K}_{f}-2{phi}_{3}\left({K}_{f}-{K}_{3}\right)}{{K}_{3}+2{K}_{f}+{phi}_{3}\left({K}_{f}-{K}_{3}\right)},$$

10 $$\frac{{\sigma }_{Tnf}}{{\sigma }_{hnf}}=\frac{{\sigma }_{1}\left(1+2{phi}_{1}\right)-{phi}_{hnf}\left(1-2{phi}_{1}\right)}{{\sigma }_{1}(1-{phi}_{1})+{\sigma }_{hnf}(1+{phi}_{1})}, \frac{{\sigma }_{hnf}}{{\sigma }_{nf}}=\frac{{\sigma }_{2}\left(1+2{phi}_{2}\right)+{\varphi }_{nf}\left(1-2{phi}_{2}\right)}{{\sigma }_{2}(1-{phi}_{2})+{\sigma }_{nf}(1+{phi}_{2})},$$

11 $$\frac{{\sigma }_{nf}}{{\sigma }_{f}}=\frac{\left({\sigma }_{3}+2{phi}_{3}{\sigma }_{3}\right)+\left({phi}_{f}-2{phi}_{3}{phi}_{f}\right)}{({\sigma }_{3}-{{\sigma }_{3}phi}_{3})+({\sigma }_{f}+{phi}_{3}{\sigma }_{f})}.$$

Dimensionless form regarding ODEs is formulated as

12 $${\left({\left|F{\prime}{\prime}\right|}^{n-1}\right)}{\prime}-\frac{{\nu }_{Thnf}}{{\nu }_{f}}\left[{\left({F}{\prime}\right)}^{2}\right]+\frac{{M}^{2}}{{\left({\beta }_{e}\right)}^{2}+{\left(1+{\beta }_{e}{\beta }_{i}\right)}^{2}}\left[{\beta }_{e}{G}{\prime}-\left(1+{\beta }_{e}{\beta }_{i}\right){F}{\prime}\right]+\frac{{A}_{1}}{{A}_{2}}{G}_{r}\Theta -\epsilon {F}{\prime}-{\mathrm{\rm A}}_{s}{F}_{r}{{F}{\prime}}^{2}-\frac{{\nu }_{Thnf}}{{\nu }_{f}}\left(\frac{2n}{n+1}F+G\right)F{\prime}{\prime}=0,$$

13 $${\left({\left|G{\prime}{\prime}\right|}^{n-1}\right)}{\prime}-\frac{{\nu }_{Thnf}}{{\nu }_{f}}{\left(G{\prime}\right)}^{2}-\frac{{M}^{2}}{{\left({\beta }_{e}\right)}^{2}+{\left(1+{\beta }_{e}{\beta }_{i}\right)}^{2}}\left[{\beta }_{e}{F}{\prime}+\left(1+{\beta }_{e}{\beta }_{i}\right){G}{\prime}\right]\frac{{A}_{1}}{{A}_{2}}{G}_{r}\Theta -\epsilon {G}{\prime}-{\mathrm{\rm A}}_{s}{F}_{r}{{G}{\prime}}^{2}-\frac{{\nu }_{Thnf}}{{\nu }_{f}}\left(\frac{2n}{n+1}F+G\right){G}^{{\prime}{\prime}}=0,$$

14 $$\left(1+{\epsilon }_{1}\Theta \right){\Theta }^{{\prime}{\prime}}+\frac{{\left(\rho {C}_{p}\right)}_{Thnf}{k}_{f}}{{\left(\rho {C}_{p}\right)}_{f}{k}_{Thnf}}\left[\left(\frac{2n}{1+n}\right)PrF{\Theta }{\prime}+PrG\Theta \right]+{\left(\frac{2n}{1+n}F+G\right)}^{2}{\Theta }^{{\prime}{\prime}}+\frac{{k}_{f}Pr\delta {Q}_{sr}}{{k}_{Thnf}}exp\left(-\delta \eta \right)+{H}_{s}Pr\left(\frac{2n}{1+n}F{\Theta }{\prime}+G\Theta {\prime}\right)+\frac{{M}^{2}}{{\left({\beta }_{e}\right)}^{2}+{\left(1+{\beta }_{e}{\beta }_{i}\right)}^{2}}\left[\frac{2n{F{\prime}}^{2}}{1+n}+{G{\prime}}^{2}\right]=0.$$

$${R}^{{\prime}{\prime}}+Sc\left(F+G\right){R}{\prime}-Sc{k}_{1}R{H}^{2}=0,$$

$$\frac{\delta }{Sc}{H}^{{\prime}{\prime}}+\left(F+G\right){H}{\prime}-{k}_{1}R{H}^{2}=0,$$

15 $$F\left(0\right)=0, {F}{\prime}\left(0\right)=1, {F}{\prime}\left(\infty \right)=0,G\left(0\right)=0, {G}{\prime}\left(0\right)=1,{G}{\prime}\left(\infty \right)=0, {R}{\prime}\left(0\right)={k}_{2}r, \delta {H}{\prime}\left(0\right)=-{k}_{2}R, R\left(\infty \right)=0, H\left(\infty \right)=0,\Theta \left(\infty \right)\to 0,\frac{{\theta }{\prime}\left(0\right)}{\gamma }=-\left(1-\theta \left(0\right)\right).$$

Flow rate in vertical and horizontal directions^51^ is prescribed as

16 $${{\left(Re\right)}^{\frac{1}{n+1}}C}_{f}=\frac{{\left(1-{phi}_{3}\right)}^{-2.5}}{{{\left(1-{phi}_{2}\right)}^{2.5}\left(1-{phi}_{1}\right)}^{2.5}}{\left|F{\prime}{\prime}(0)\right|}^{n},$$

17 $${{\left(Re\right)}^{\frac{1}{n+1}}C}_{g}=\frac{{\left(1-{phi}_{3}\right)}^{-2.5}}{{{\left(1-{phi}_{2}\right)}^{2.5}\left(1-{phi}_{1}\right)}^{2.5}}{\left|G{\prime}{\prime}(0)\right|}^{n}.$$

Nusselt number^51^ is

18 $$Nu{\left(Re\right)}^{\frac{-1}{n+1}}=\frac{{k}_{thnf}}{{k}_{f}}{\theta }{\prime}\left(0\right).$$

## Numerical procedure

### Derivation of residuals

Terms of all dimensionless equations are collected on one side and applications of indigitation are implemented. Weight functions are multiplied with whole process termed as residuals. The residuals are defined as

19 $${\int }_{{\eta }_{e}}^{{\eta }_{e+1}}{w}_{1}\left[{F}{\prime}-O\right]d\eta =0,{\int }_{{\eta }_{e}}^{{\eta }_{e+1}}{w}_{2}\left[{G}{\prime}-P\right]d\eta =0,$$

20 $${\int }_{{\eta }_{e}}^{{\eta }_{e+1}}{w}_{3}\left[\begin{array}{c}{\left({\left|{O}{\prime}\right|}^{n-1}\right)}{\prime}-\frac{{\nu }_{Thnf}}{{\nu }_{f}}\left[{\left(O\right)}^{2}\right]+\frac{{M}^{2}}{{\left({\beta }_{e}\right)}^{2}+{\left(1+{\beta }_{e}{\beta }_{i}\right)}^{2}}\left({\beta }_{e}P-\left(1+{\beta }_{e}{\beta }_{i}\right)O\right)\\ +\frac{{A}_{1}}{{A}_{2}}{G}_{r}\Theta -\epsilon O-{\mathrm{\rm A}}_{s}{F}_{r}{O}^{2}-\frac{{\nu }_{Thnf}}{{\nu }_{f}}\left(\frac{2n}{n+1}F+G\right)O{\prime}\end{array}\right]d\eta =0,$$

21 $${\int }_{{\eta }_{e}}^{{\eta }_{e+1}}{w}_{4}\left[\begin{array}{c}{\left({\left|P{\prime}\right|}^{n-1}\right)}{\prime}-\frac{{\nu }_{Thnf}}{{\nu }_{f}}{\left(P\right)}^{2}-\frac{{M}^{2}}{{\left({\beta }_{e}\right)}^{2}+{\left(1+{\beta }_{e}{\beta }_{i}\right)}^{2}}\left[{\beta }_{e}O+\left(1+{\beta }_{e}{\beta }_{i}\right)P\right]\\ \frac{{A}_{1}}{{A}_{2}}{G}_{r}\Theta -\epsilon P-{\mathrm{\rm A}}_{s}{F}_{r}{P}^{2}-\frac{{\nu }_{Thnf}}{{\nu }_{f}}\left(\frac{2n}{n+1}F+G\right)P{\prime}\end{array}\right]d\eta =0,$$

22 $${\int }_{{\eta }_{e}}^{{\eta }_{e+1}}{w}_{5}\left[\begin{array}{c}\left(1+{\epsilon }_{1}\Theta \right){\Theta }^{{\prime}{\prime}}+\frac{{\left(\rho {C}_{p}\right)}_{Thnf}{k}_{f}}{{\left(\rho {C}_{p}\right)}_{f}{k}_{Thnf}}\left[\left(\frac{2n}{1+n}\right)PrF{\Theta }{\prime}+PrG\Theta \right]\\ \frac{{k}_{f}Pr\delta {Q}_{sr}}{{k}_{Thnf}}exp\left(-\delta \eta \right)++{\left(\frac{2n}{1+n}F+G\right)}^{2}{\Theta }^{{\prime}{\prime}}\\ +{H}_{s}Pr\left(\frac{2n}{1+n}F{\Theta }{\prime}+G\Theta {\prime}\right)+\frac{{M}^{2}}{{\left({\beta }_{e}\right)}^{2}+{\left(1+{\beta }_{e}{\beta }_{i}\right)}^{2}}\left[\frac{2n{P}^{2}}{1+n}+{P}^{2}\right]\end{array}\right]d\eta =0,$$

23 $${\int }_{{\eta }_{e}}^{{\eta }_{e+1}}{w}_{6}\left[{R}^{{\prime}{\prime}}+Sc\left(F+G\right){R}{\prime}-Sc{k}_{1}R{H}^{2}\right]d\eta =0,$$

24 $${\int }_{{\eta }_{e}}^{{\eta }_{e+1}}{w}_{7}\left[\frac{\delta }{Sc}{H}^{{\prime}{\prime}}+\left(F+G\right){H}{\prime}-{k}_{1}R{H}^{2}\right]d\eta =0.$$

Here, ${w}_{i} \left(i=1..7\right)$ are termed as weighted functions. Unknowns and shape functions are defined as

25 $$O=\sum_{j=1}^{2}{O}_{i}{\psi }_{j}, P=\sum_{j=1}^{2}{P}_{i}{\psi }_{j}, G=\sum_{j=1}^{2}{G}_{i}{\psi }_{j}, F=\sum_{j=1}^{2}{F}_{i}{\psi }_{j},\Theta =\sum_{j=1}^{2}{\Theta }_{i}{\psi }_{j},$$

26 $${\psi }_{j}={\left(-1\right)}^{i-1}\left(\frac{\eta -{\eta }_{j-1}}{{\eta }_{i}-{\eta }_{j-1}}\right), R=\sum_{j=1}^{2}{R}_{i}{\psi }_{j}, H=\sum_{j=1}^{2}{H}_{i}{\psi }_{j}.$$

### Weak forms

Strong form (dimensionless ODEs) is transformed into weak forms using concept of residuals. After it, stiffness elements have been obtained.

### Galerkin approximations

Galerkin approximations have been utilized to produce stiffness elements and weak forms. Global stiff matrix has been also achieved. Stiffness matrices are

27 $${{K}_{ij}^{13}=-{\int }_{{\eta }_{e}}^{{\eta }_{e+1}}{\psi }_{i}\left(\frac{d{\psi }_{j}}{d\eta }\right)d\eta , {K}_{ij}^{12}=0, K}_{ij}^{11}={\int }_{{\eta }_{e}}^{{\eta }_{e+1}}{\psi }_{i}\left(\frac{d{\psi }_{j}}{d\eta }\right)d\eta , {K}_{ij}^{14}=0,$$

28 $${K}_{ij}^{24}=-{\int }_{{\eta }_{e}}^{{\eta }_{e+1}}{\psi }_{i}\left(\frac{d{\psi }_{j}}{d\eta }\right)d\eta , {K}_{ij}^{22}= {K}_{ij}^{23}={K}_{ij}^{26}={K}_{ij}^{25}=0,{K}_{ij}^{26}=0,$$

29 $${K}_{ij}^{21}={\int }_{{\eta }_{e}}^{{\eta }_{e+1}}{\psi }_{i}\left(\frac{d{\psi }_{j}}{d\eta }\right)d\eta , {K}_{ij}^{27}=0, {K}_{ij}^{15}=0, {K}_{ij}^{16}=0, {K}_{ij}^{17}=0, {K}_{ij}^{34}=-{\int }_{{\eta }_{e}}^{{\eta }_{e+1}}{\frac{{A}_{1}}{{A}_{2}}{{\psi }_{j}G}_{r}\psi }_{i}d\eta ,$$

30 $${K}_{ij}^{33}={\int }_{{\eta }_{e}}^{{\eta }_{e+1}}\left[\begin{array}{c}-\left(n-1\right){\left(\overline{{O }{\prime}}\right)}^{n-1}\frac{d{\psi }_{i}}{d\eta }\frac{d{\psi }_{j}}{d\eta }-\frac{{\nu }_{Thnf}}{{\nu }_{f}}\overline{O}{\psi }_{i}{\psi }_{j}+\frac{{M}^{2}{\beta }_{e}}{{\left({\beta }_{e}\right)}^{2}+{\left(1+{\beta }_{e}{\beta }_{i}\right)}^{2}}{\psi }_{i}{\psi }_{j}\\ -\frac{{M}^{2}\left(1+{\beta }_{e}{\beta }_{i}\right)}{{\left({\beta }_{e}\right)}^{2}+{\left(1+{\beta }_{e}{\beta }_{i}\right)}^{2}}{\psi }_{i}{\psi }_{j}-\epsilon {\psi }_{i}{\psi }_{j}--{\mathrm{\rm A}}_{s}{F}_{r}\overline{O}{\psi }_{i}{\psi }_{j}\\ -\frac{{\nu }_{Thnf}}{{\nu }_{f}}\left(\frac{2n}{n+1}\left(\overline{F }+\overline{G }\right)\right){\psi }_{j}\frac{d{\psi }_{j}}{d\eta }\end{array}\right]d\eta ,$$

31 $${K}_{ij}^{31}=0, {K}_{ij}^{33}=0, {K}_{ij}^{35}=0, {K}_{ij}^{36}=0, {K}_{ij}^{37}=0.$$

32 $${K}_{ij}^{44}={\int }_{{\eta }_{e}}^{{\eta }_{e+1}}\left[\begin{array}{c}-\left(n-1\right){\left(\overline{{P }{\prime}}\right)}^{n-1}\frac{d{\psi }_{i}}{d\eta }\frac{d{\psi }_{j}}{d\eta }-\frac{{M}^{2}{\beta }_{e}{\psi }_{j}{\psi }_{i}}{{\left({\beta }_{e}\right)}^{2}+{\left(1+{\beta }_{e}{\beta }_{i}\right)}^{2}}-\epsilon {\psi }_{j}{\psi }_{i}\\ -\frac{{M}^{2}\left(1+{\beta }_{e}{\beta }_{i}\right)}{{\left({\beta }_{e}\right)}^{2}+{\left(1+{\beta }_{e}{\beta }_{i}\right)}^{2}}{\psi }_{j}{\psi }_{i}-{\psi }_{i}{\psi }_{j}{\mathrm{\rm A}}_{s}{F}_{r}\overline{P }-\frac{{\nu }_{Thnf}}{{\nu }_{f}}\left(\frac{2n}{n+1}\overline{F }+\overline{G }\right){\psi }_{j}{\psi }_{i}\end{array}\right]d\eta ,$$

33 $${K}_{ij}^{44}=-{\int }_{{\eta }_{e}}^{{\eta }_{e+1}}{\frac{{A}_{1}}{{A}_{2}}{{\psi }_{j}G}_{r}\psi }_{i}d\eta , {K}_{ij}^{41}=0, {K}_{ij}^{42}=0, {K}_{ij}^{45}=0, {K}_{ij}^{46}=0, {K}_{ij}^{47}=0,$$

34 $${K}_{ij}^{55}=-{\int }_{{\eta }_{e}}^{{\eta }_{e+1}}\left[\begin{array}{c}-\left(1+{\epsilon }_{1}\Theta \right)\frac{d{\psi }_{i}}{d\eta }\frac{d{\psi }_{j}}{d\eta }+\frac{{\left(\rho {C}_{p}\right)}_{Thnf}{k}_{f}}{{\left(\rho {C}_{p}\right)}_{f}{k}_{Thnf}}\left(\frac{2n}{1+n}\right)PrF{\psi }_{i}\frac{d{\psi }_{j}}{d\eta }\\ \frac{{\left(\rho {C}_{p}\right)}_{Thnf}{k}_{f}}{{\left(\rho {C}_{p}\right)}_{f}{k}_{Thnf}}Pr\overline{G}{\psi }_{i}{\psi }_{j}+\frac{{k}_{f}Pr\delta {Q}_{sr}}{{k}_{Thnf}}exp\left(-\delta \eta \right)+{\left(\frac{2n}{1+n}\overline{F }+\overline{G }\right)}^{2}\frac{d{\psi }_{i}}{d\eta }\frac{d{\psi }_{j}}{d\eta }\\ +{H}_{s}Pr\left(\frac{2n}{1+n}F{\Theta }{\prime}+G\Theta {\prime}\right)\end{array}\right]d\eta ,$$

35 $${K}_{ij}^{52}={\int }_{{\eta }_{e}}^{{\eta }_{e+1}}\left[\frac{{M}^{2}}{{\left({\beta }_{e}\right)}^{2}+{\left(1+{\beta }_{e}{\beta }_{i}\right)}^{2}}\left[\frac{2n\overline{P} }{1+n}{\psi }_{i}{\psi }_{j}+{\psi }_{i}{\psi }_{j}\overline{P }\right]\right]d\eta , {K}_{ij}^{51}=0, {K}_{ij}^{52}=0, {K}_{ij}^{53}=0,$$

36 $${K}_{ij}^{66}=-{\int }_{{\eta }_{e}}^{{\eta }_{e+1}}\left[-\frac{d{\psi }_{i}}{d\eta }\frac{d{\psi }_{j}}{d\eta }+Sc\left(\overline{F }+\overline{G }\right){\psi }_{i}\frac{d{\psi }_{j}}{d\eta }-Sc{k}_{1}R\overline{H}{\psi }_{j}{\psi }_{i}\right]d\eta , {K}_{ij}^{54}=0, {K}_{ij}^{55}=0,$$

37 $${K}_{ij}^{77}=-{\int }_{{\eta }_{e}}^{{\eta }_{e+1}}\left[-\frac{\delta }{Sc}\frac{d{\psi }_{i}}{d\eta }\frac{d{\psi }_{j}}{d\eta }+\left(\overline{F }+\overline{G }\right){\psi }_{i}\frac{d{\psi }_{j}}{d\eta }-{k}_{1}R\overline{H}{\psi }_{j}{\psi }_{i}\right]d\eta , {K}_{ij}^{56}=0, {K}_{ij}^{57}=0,$$

38 $${K}_{ij}^{71}=0, {K}_{ij}^{72}=0, {K}_{ij}^{73}=0, {K}_{ij}^{74}=0, {K}_{ij}^{56}=0, {K}_{ij}^{75}=0, {K}_{ij}^{56}=0, {K}_{ij}^{76}=0, {K}_{ij}^{56}=0, {K}_{ij}^{77}=0.$$

### Assembly development and algebraic equations

A developed system of algebraic equations was obtained utilizing assembly process. After it, Picard linearization is implemented to derive system of algebraic (linear). An error investigation is

39 $$Err=\left|{\chi }^{i}-{\chi }^{i-1}\right|, Max\left|{\chi }^{i}-{\chi }^{i-1}\right|\le {10}^{-8}.$$

### Mesh-free

Grid sizes are derived in Table 2 while convergence has been ensured considering domain [0, 8].

**Table 2:** Grid independent investigation of $H\left(\frac{{\eta }_{max}}{2}\right), R\left(\frac{{\eta }_{max}}{2}\right), F{\prime}\left(\frac{{\eta }_{max}}{2}\right)$ and $G{\prime}\left(\frac{{\eta }_{max}}{2}\right)$.

$e$	$F{\prime}\left(\frac{{\eta }_{max}}{2}\right)$	$G{\prime}\left(\frac{{\eta }_{max}}{2}\right)$	$\theta \left(\frac{{\eta }_{max}}{2}\right)$	$H\left(\frac{{\eta }_{max}}{2}\right)$	$R\left(\frac{{\eta }_{max}}{2}\right)$
15	0.5749398874	0.5762083625	0.728320671	0.348748040	0.342976482
30	0.5649398874	0.5662083625	0.621021530	0.342976482	0.348748040
60	0.5486333822	0.5544666263	0.620304009	0.216581180	0.245401724
90	0.5303775409	0.5538372465	0.613047073	0.206489869	0.818808599
120	0.5212133714	0.5523084157	0.552938251	0.181502751	0.641984429
150	0.5139820934	0.5520020171	0.542956932	0.173292605	0.623394291
180	0.5106482296	0.5406549903	0.543998456	0.618636037	0.618670712
210	0.5086640344	0.5486669638	0.532399058	0.617220336	0.617235412
240	0.5086885457	0.5376894120	0.532562217	0.603954399	0.203963716
270	0.5085906791	0.5373615048	0.532729481	0.617379749	0.617084015
300	0.5085946401	0.5373251379	0.533252247	0.617390637	0.617393210

## Outcomes and discussion

Three-dimensional model for power law model is generated including impacts of Hall currents. It is importantly mentioned that 3 types of nanoparticles are imposed in the suspension of ethylene glycol. Mechanism of heat sink and heat source are implemented in heat energy mechanism. Furthermore, Joule heating is also studied. Such complex model is solved using numerical scheme called finite element method. Detail discussion regarding thermal and velocity profiles are mentioned below.

### Dynamical behavior related to fluidic motion

**Figure 3:**
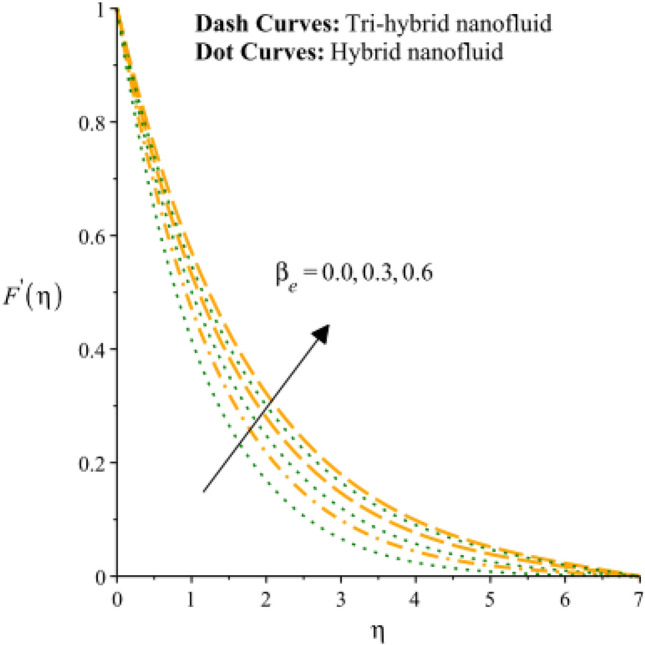
Variation of velocity curves in x-direction against ${\beta }_{e}.$

**Figure 4:**
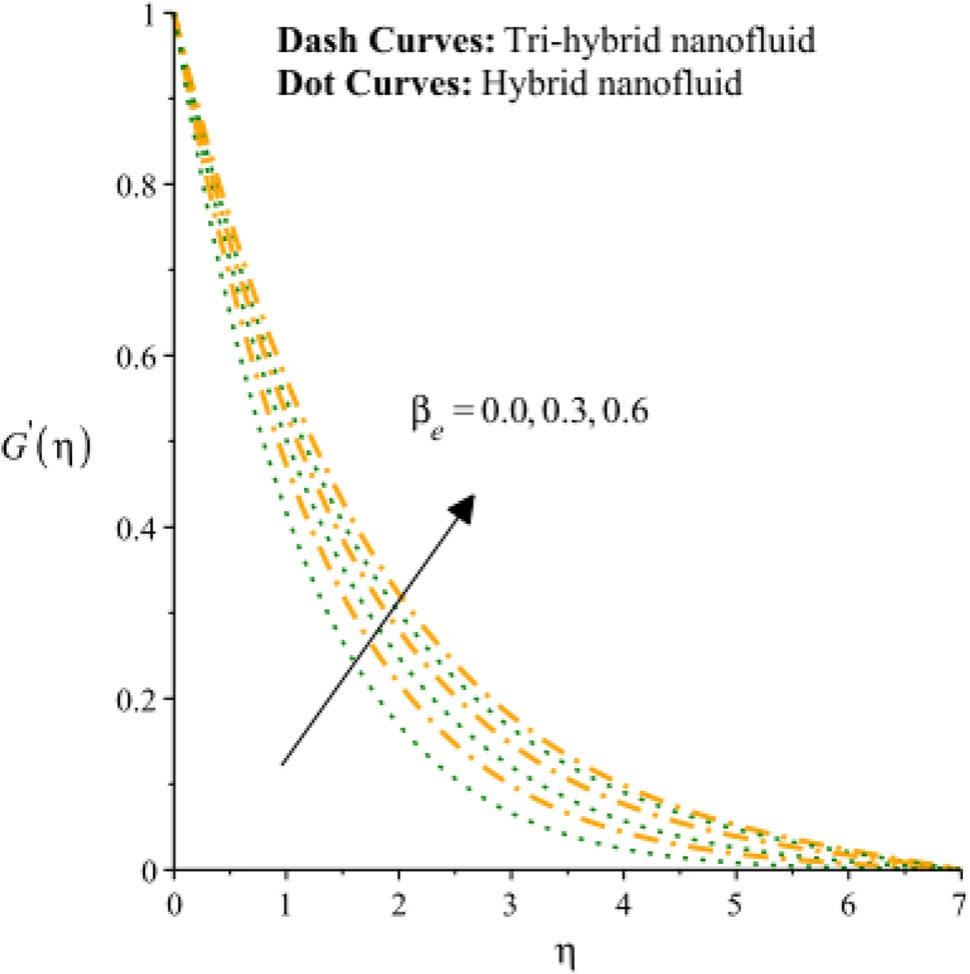
Variation of curves in y-direction against ${\beta }_{e}.$

**Figure 5:**
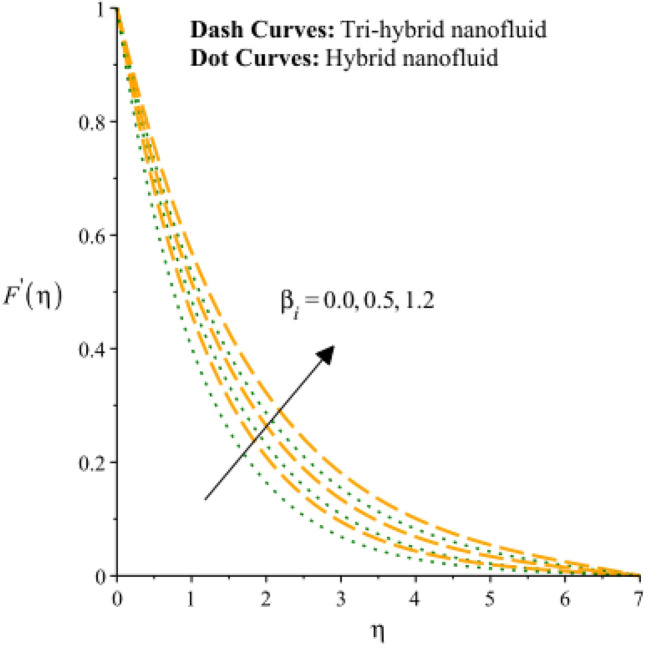
Variation of velocity curves in x-direction against ${\beta }_{i}.$

**Figure 6:**
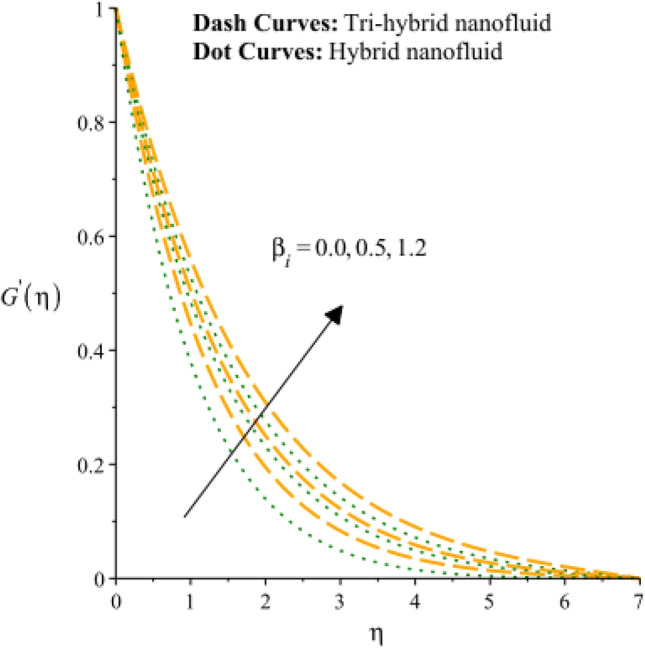
Variation of velocity curves in y-direction against ${\beta }_{i}.$

**Figure 7:**
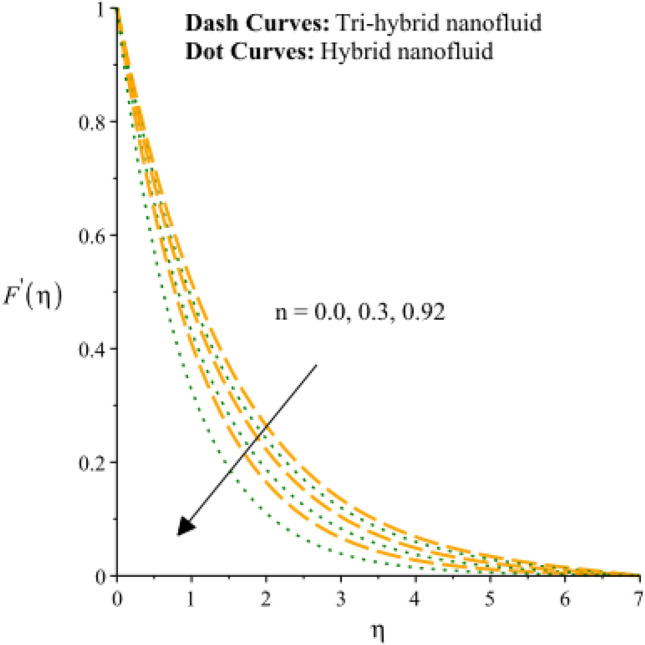
Variation of velocity curves in x-direction against $n.$

**Figure 8:**
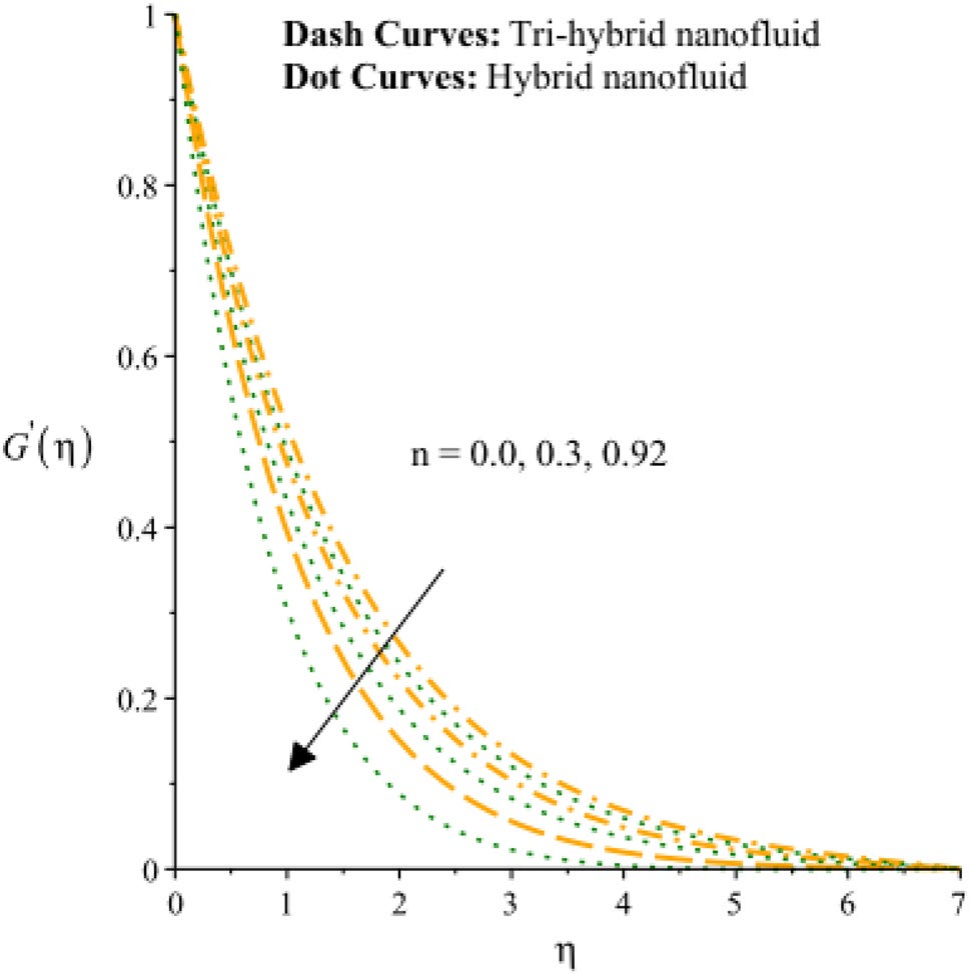
Variation of velocity curves in y-direction against $n.$

**Figure 9:**
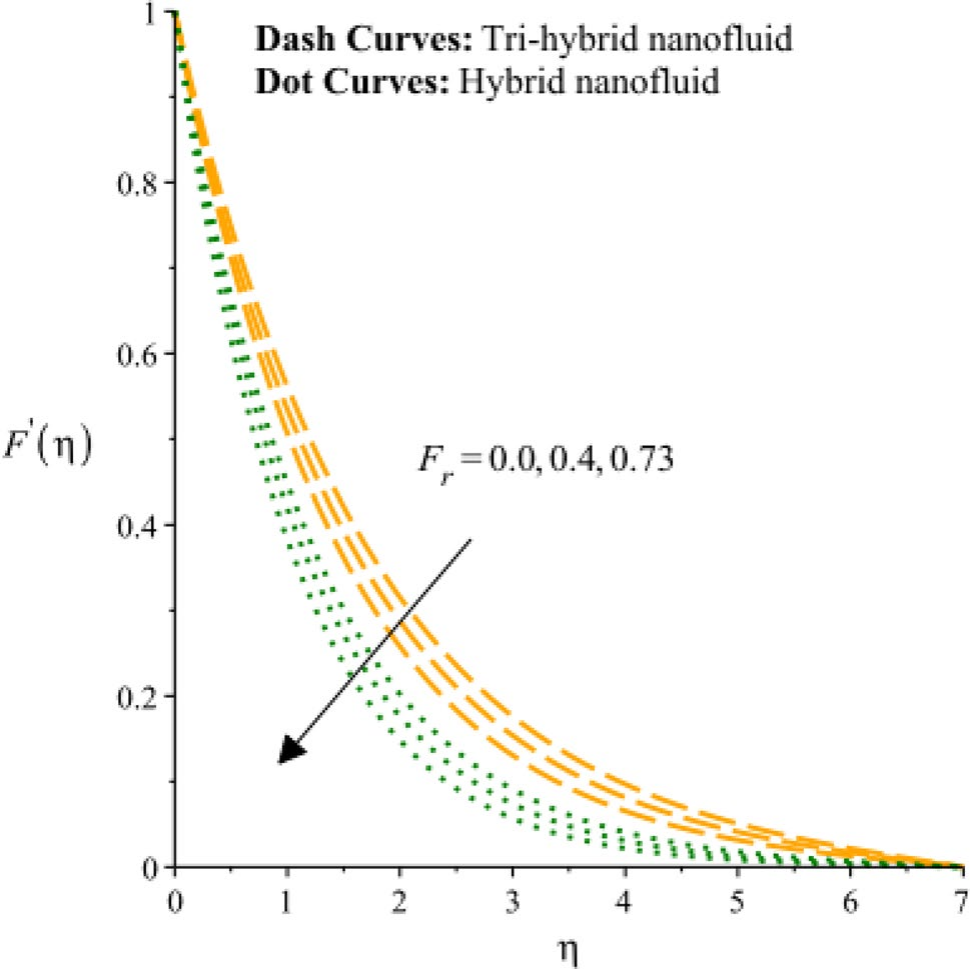
Variation of velocity curves in x-direction against ${F}_{r}.$

**Figure 10:**
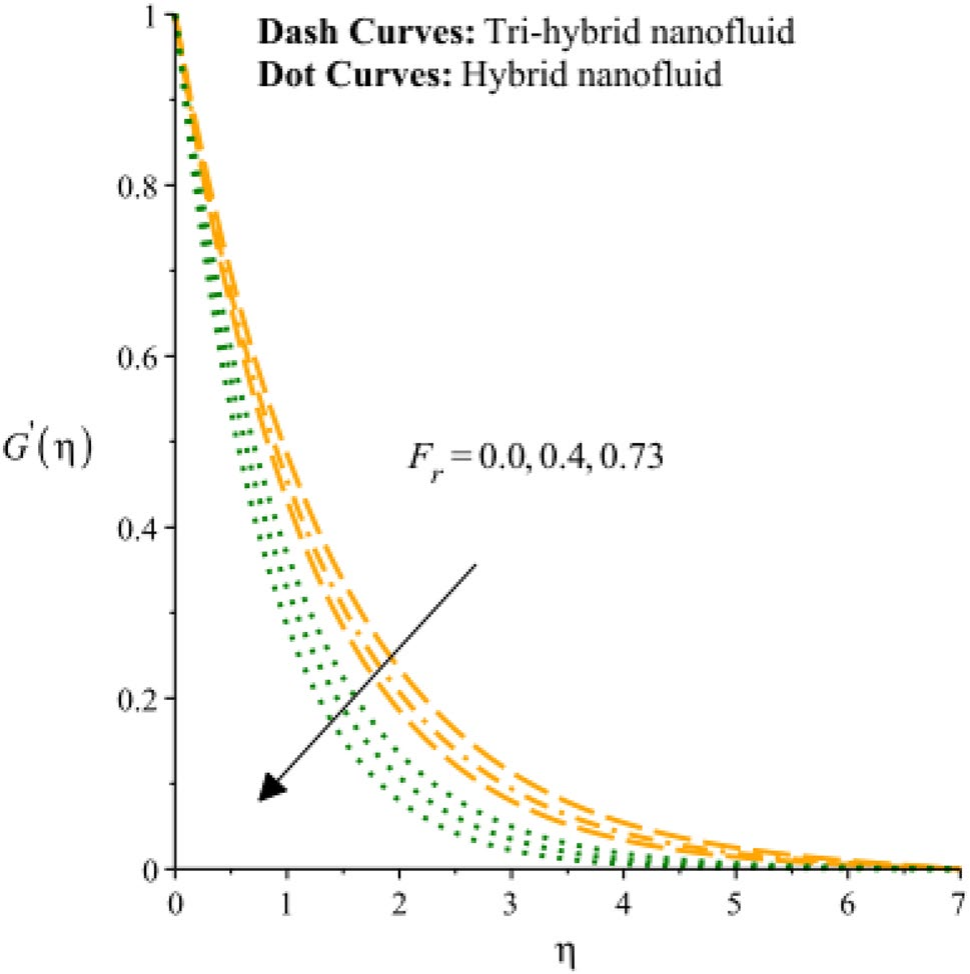
Variation of velocity curves in y-direction against ${F}_{r}.$

**Figure 11:**
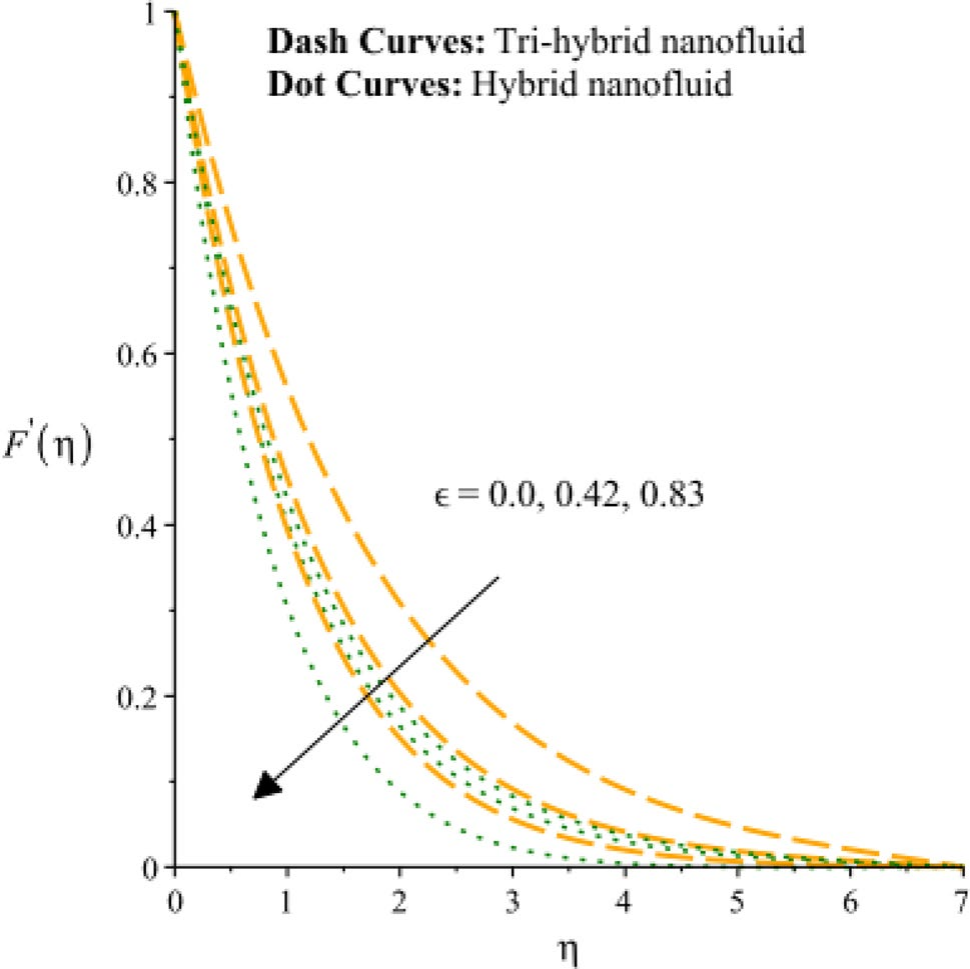
Variation of velocity curves in x-direction against $\epsilon .$

**Figure 12:**
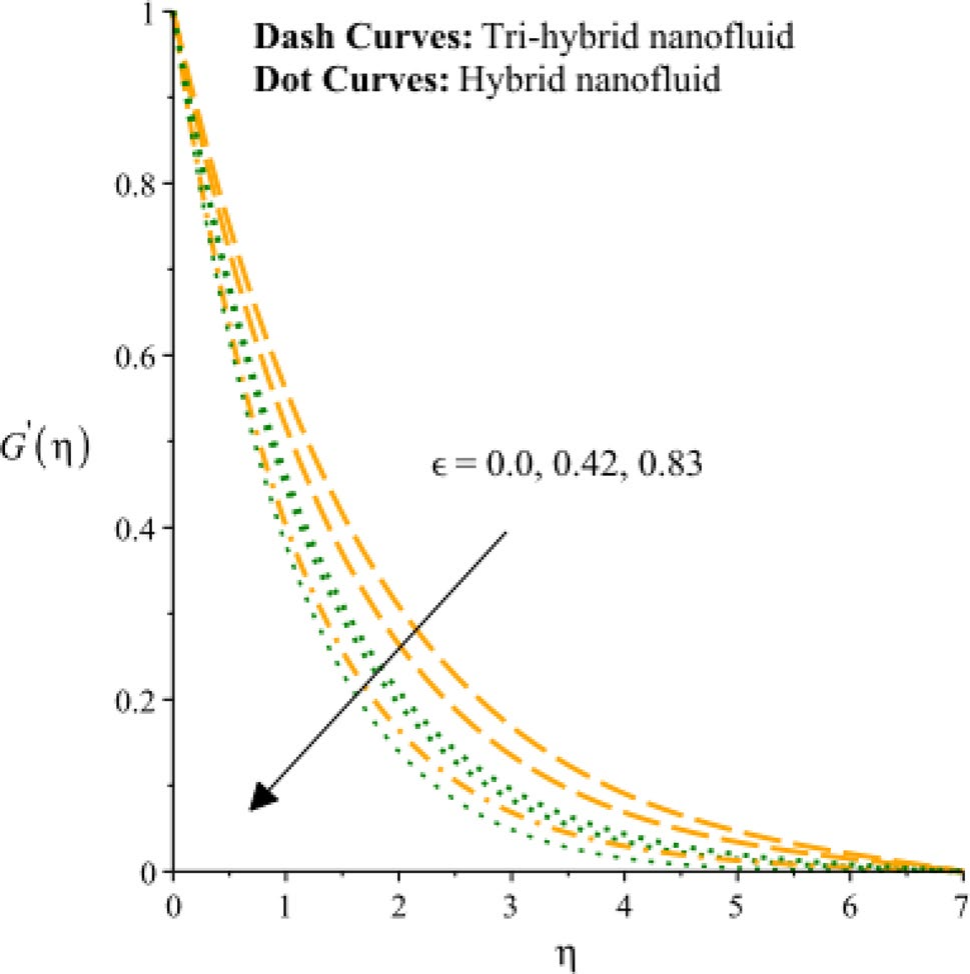
Variation of velocity curves in y-direction against $\epsilon .$

Figures 3, 4, 5, 6, 7 and 8 are plotted to notify behavior of ${\beta }_{e}$, ${\beta }_{i}$ and $n$ on velocity profiles in view of y- and x-directions inclusion of composite of nanomaterial. It was estimated that solid lines are drawn for visualization tri-hybrid nanomaterial and dot lines are prescribed the impact of hybrid nanomaterial. Figures 3 and 4 are plotted to notice the relationship among fluidic motion and Hall parameter. The acceleration into fluidic particles is enhanced when Hall parameter is inclined. It is observed that appearance of Hall force is produced because of generalize Ohm’s law. Moreover, it is included that acceleration is higher than fluidic motion for hybrid nanomaterial. A characteristic parameter used to characterize how charged particles behave in a magnetic field is the hall parameter, also known as the hall coefficient or hall parameter constant. It is essential to the Hall effect, which is the phenomenon of moving charged particles being deflected through a conductor in a direction opposite to both the magnetic field and the current flow. The bigger the magnetic field or the electric field perpendicular to the current density must be when the Hall parameter is raised. This shows that the magnetic field's effect on the charged particles’ force is also escalating. Figures 5 and 6 are captured to find impact of ion slip number on velocity components (in y- and x-directions). From these figures, same impact for ion slip number is accumulated on fluidic motion. Because, frictional force among fluidic particles is observed as a negligible when ion slip number is enhanced. Therefore, viscosity of fluidic particles past a surface becomes declined. Hence, acceleration was magnified versus incline values of ${\beta }_{i}$. The behavior of ions in a plasma or fluid flow is described by the ion slip parameter, which is a characteristic parameter. It shows how fast the ions are moving in comparison to neutral particles or the surrounding fluid. The ion velocity is greater in comparison to the mean neutral particle velocity when the ion slip parameter is raised. The ratio of ion velocity to neutral particle velocity increases as a result. The increased velocity differential between the ions and the surrounding particles causes the velocity field to expand as a result. Role of power law index number is addressed on velocity curves. It is visualized that occurrence of $n$ is created when power law material is utilized as depicted through Figs. 7 And 8. From graphical view, motion of fluidic particles become reduce when $n$ is increased. The behavior of a velocity field is described by the $n$. In a power law connection between the magnitude of the velocity and the position inside the flow field, it serves as the exponent. Increases in the power law index parameter translate into increases in the exponent $n$. As a result, the velocity field deviates from the flow field more quickly with position. By taking into account the power law expression, this may be mathematically observed. Therefore, thickness along with momentum layers is reduced versus incline values of power law number which are shown in Figs. 7 And 8. Further, tri-hybrid nanomaterial is observed as significant on flow impact as compared for hybrid nanomaterial. Figures 9 and 10 are revealed motion regarding ternary nanoparticles with change in ${F}_{r}.$ It was experienced that motion associated with tri-hybrid nanoparticles become slow down when ${F}_{r}$ is declined. Variation of vecloity curve with different values of $\epsilon$ is deducted by Figs. 11 And 12. Similar impact has been investigated into motion of tri-hybrid nanoparticles versus change in $\epsilon .$ A characteristic parameter used to characterize the behavior of fluid flow through a porous media is the porosity parameter. A bigger percentage of unoccupied space is implied on a surface by a rise in the porosity parameter, which causes the velocity field to drop. This has a mathematical connection to the medium's permeability. Porosity and permeability are inversely correlated, therefore an increase in porosity results in a decrease in permeability. Darcy's law states that the permeability is directly proportional to the velocity field.

### Dynamical behavior related to thermally mechanism

**Figure 13:**
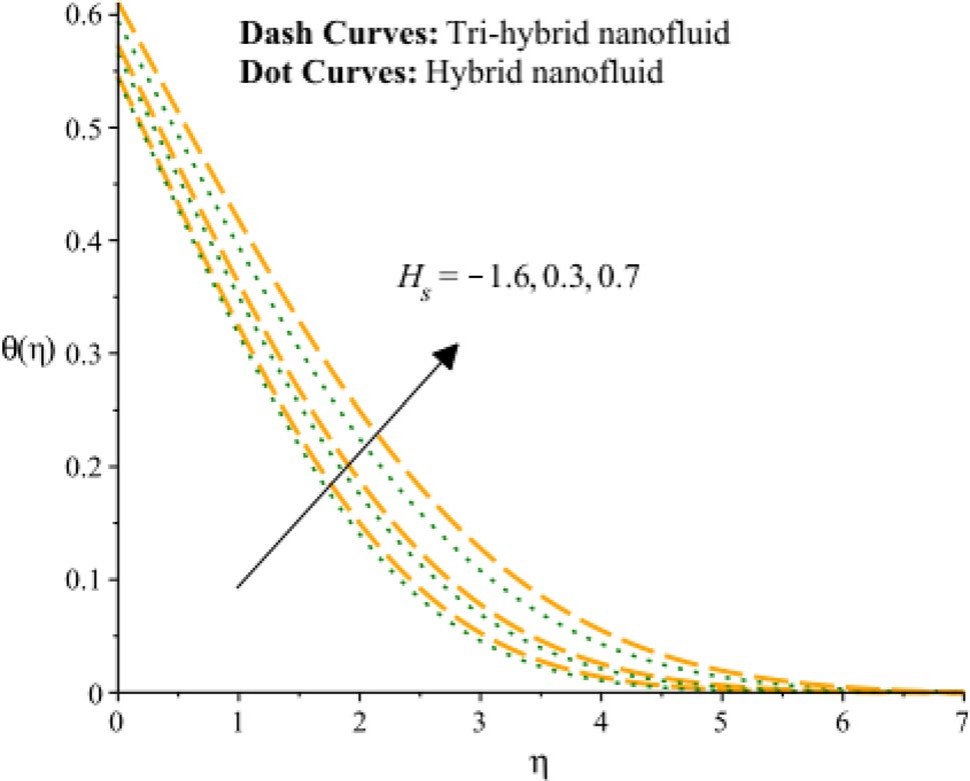
Variation of temperature curves against ${H}_{s}.$

**Figure 14:**
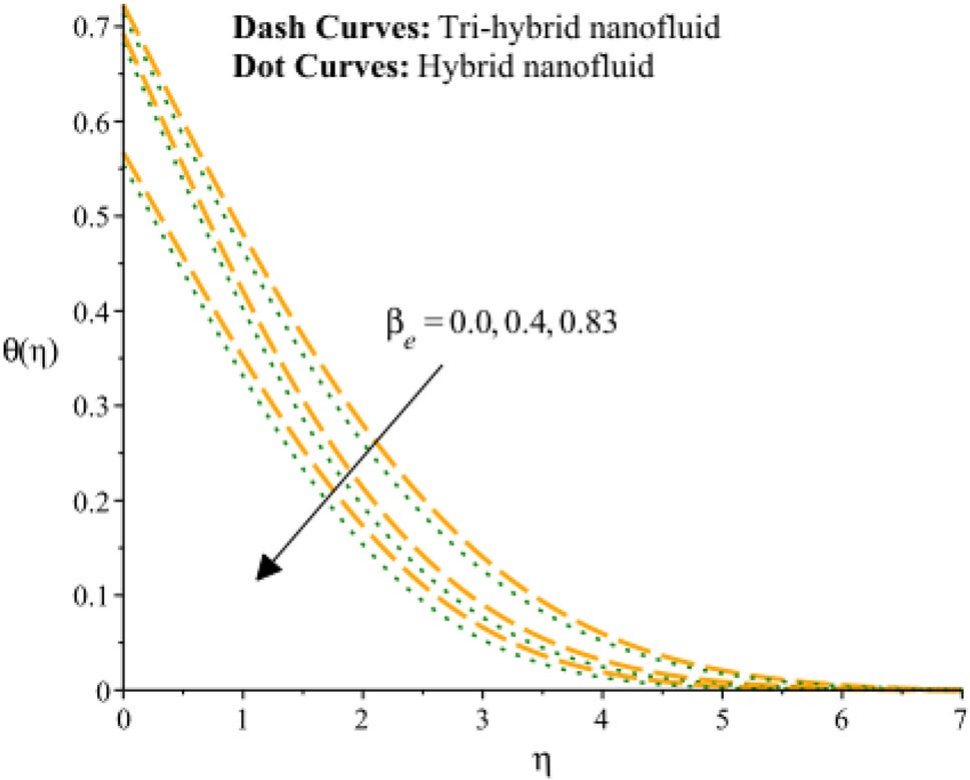
Variation of temperature curves against ${\beta }_{e}.$

**Figure 15:**
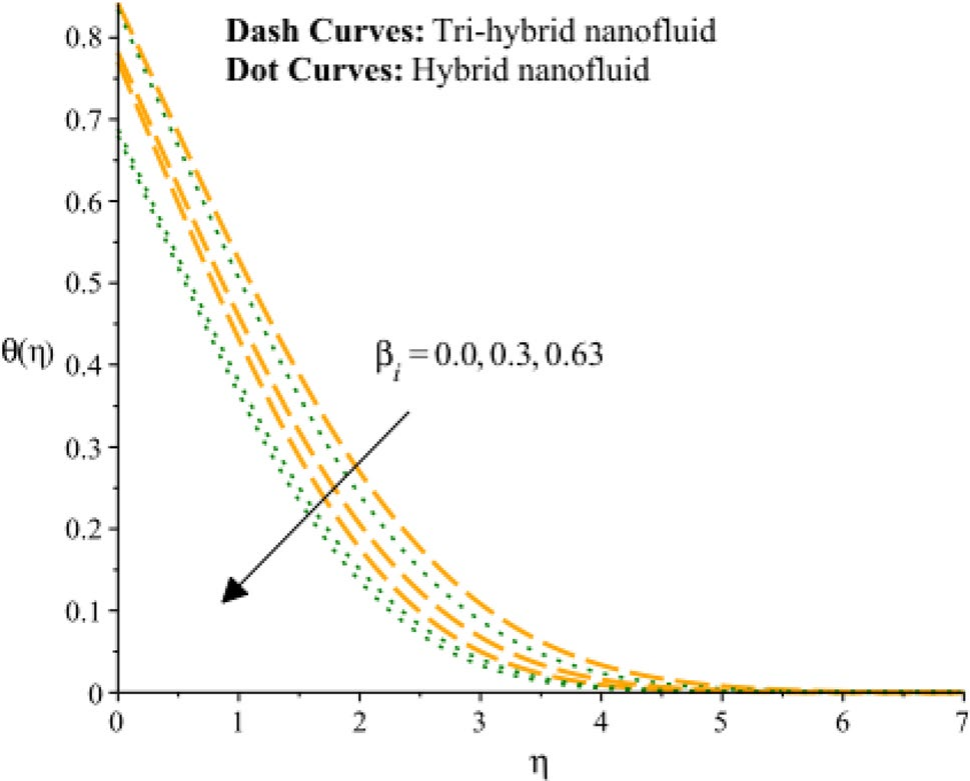
Variation of temperature curves against ${\beta }_{i}.$

**Figure 16:**
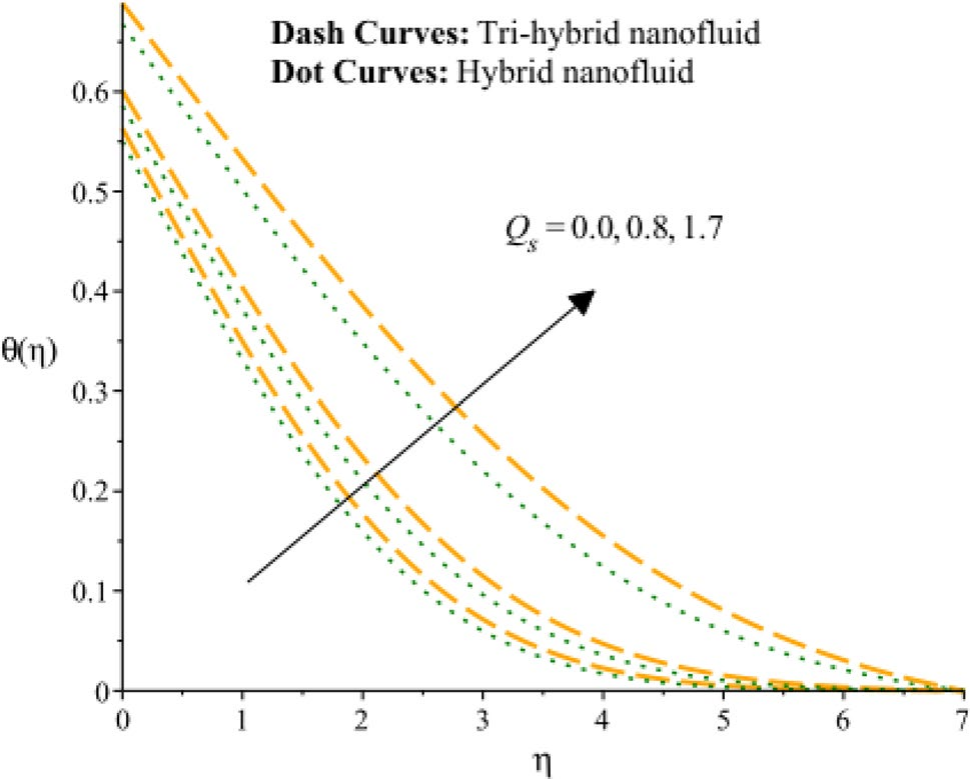
Variation of temperature curves against ${Q}_{s}.$

**Figure 17:**
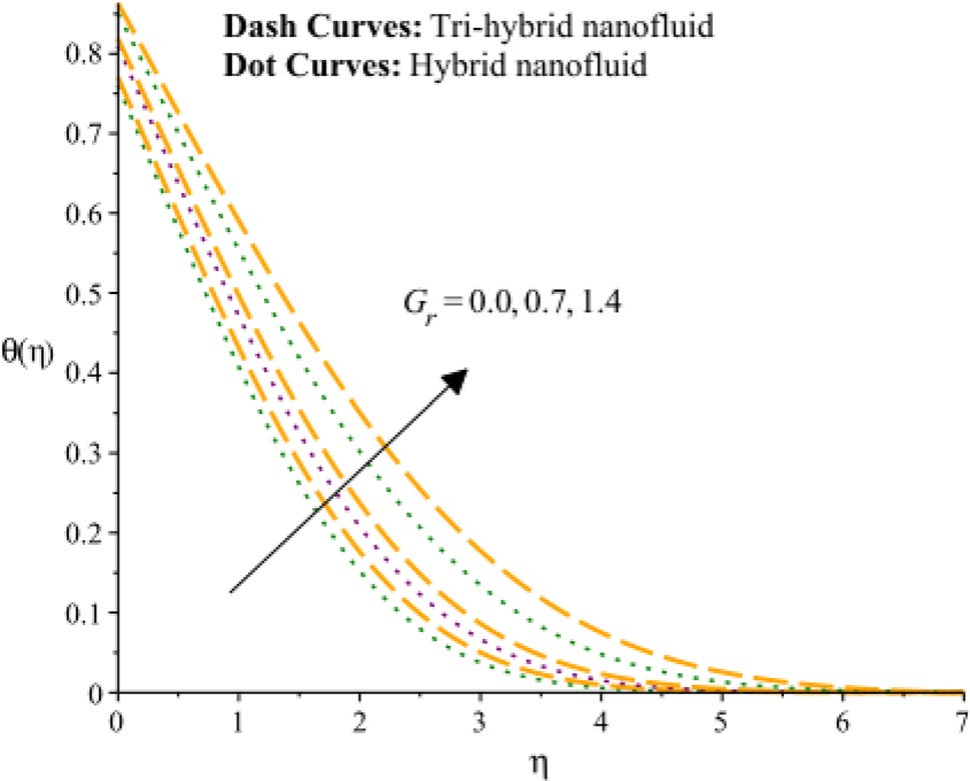
Variation of temperature curves against ${G}_{r}.$

Figures 13, 14 and 15 are developed for estimation of temperature against the change in ${H}_{s}$. Figure 9 predicts observation of ${H}_{s}$ on heat energy. It is predicted that maximum production related to thermal energy is addressed considering an impact of ${H}_{s}$. Mathematically, heat source number has directly proportion relation against temperature difference. Therefore, amount regarding temperature can be adjusted using variation in ${H}_{s}$. The amount of the heat source component in the equation for heat conduction increases when the heat source parameter is raised. Mathematically, this results in a higher rate of heat generation or addition inside the system. Increases in the heat source parameter indicate that more heat is being supplied to the surface. The surface’s total energy is increased by the extra heat, which raises temperatures. Moreover, TBLT for tri-hybrid nanomaterial is higher than for hybrid nanomaterial. Figures 10 and 11 are plotted to capture an influence of Hall parameter and ${\beta }_{i}$ on temperature profile. Fluidic heat energy is significantly decreased when ion slip and Hall numbers are magnified. It is occurred because ${\beta }_{e}$ and ${\beta }_{i}$ was formulated in the presence of generalized Ohm’s law. Mathematically, sum of ${\beta }_{e}$ and ${\beta }_{i}$ is appeared in denominator in Joule heating term. Therefore, inversely proportional relation is observed among partially ionized particles and heat energy. Hence, an increment in ${\beta }_{e}$ and ${\beta }_{i}$ and heat energy is reduced. The Hall parameter characterizes how charged particles behave in a magnetic field. The ratio of the electric field perpendicular to current flow to the sum of the magnetic field and current density is represented by the Hall parameter. The Hall effect, which is the deflection of moving charged particles in a conductor caused by the combined impact of magnetic and electric fields, is measured by the intensity of the effect. Instead of affecting the temperature field, the Hall effect largely impacts the mobility of charged particles and the consequent electrical activity. While an increase in the ion slip parameter may result in modifications to the overall fluid flow or electrical behavior, it directly affects the temperature field-determining heat transfer processes. Therefore, there is physical evidence to support the hypothesis that a drop in the temperature field would follow an increase in the ion slip parameter. Figure 16 experiences the role of ${Q}_{s}$ on temperature curve and inclination is observed by enhancing values of ${Q}_{s}.$ Solar thermal radiation, or energy emitted by the Sun in the form of electromagnetic radiation, has the potential to affect the temperature field. The Stefan-Boltzmann law may be used to explain the mathematical link between the temperature field and the solar thermal radiation parameter. The incident solar thermal radiation on the surface increases as the solar thermal radiation parameter increases. The power radiated in the equation rises as a result. As a result, the temperature must rise in order to preserve energy balance. As a result, the temperature field increases as the solar thermal radiation parameter increases, according to mathematics. An increasing trend on temperature curve versus buoyancy parameter (${G}_{r}$) is addressed by Fig. 17. This increasing impact occurred due to influence of gravity on the fluid medium is the physical cause of the rise in temperature field when the gravitational force is increased. The driving factor behind fluid motion and the transmission of energy is gravity.

### Dynamical behavior related to reactions of concentration rate

**Figure 18:**
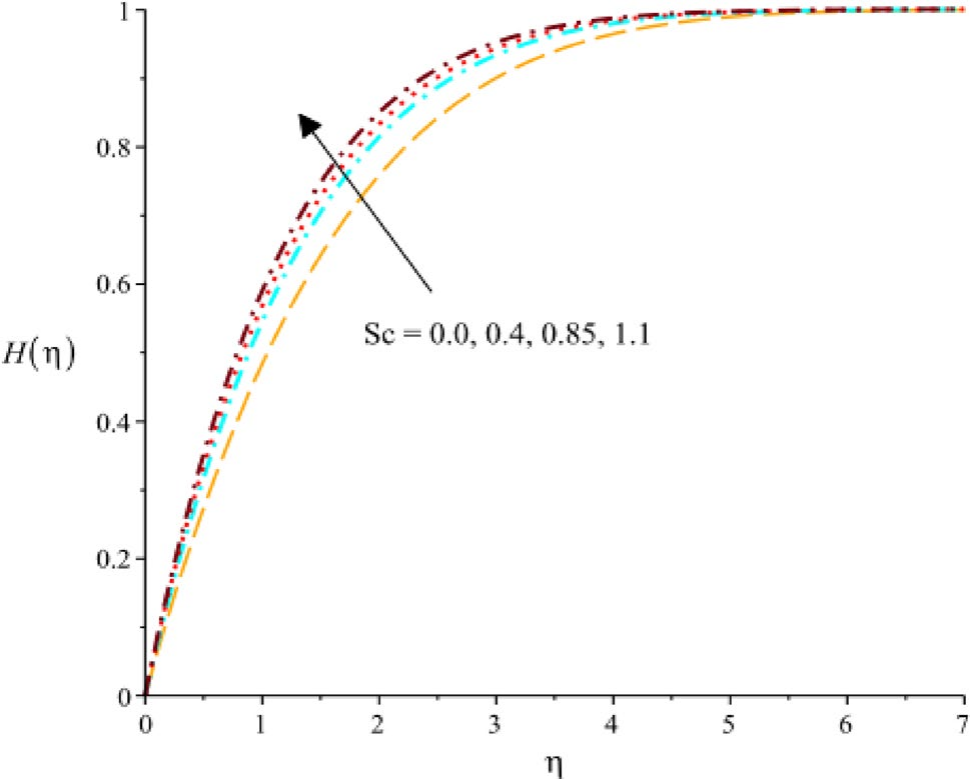
Variation of $H(\eta )$ against $Sc.$

**Figure 19:**
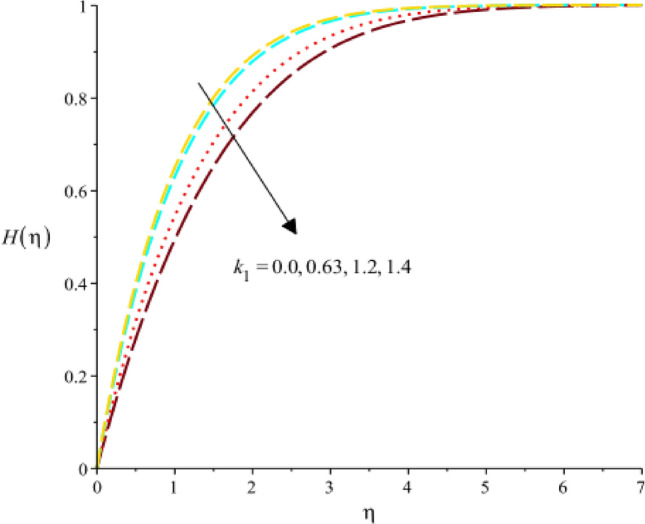
Variation of $H(\eta )$ against ${k}_{1}.$

**Figure 20:**
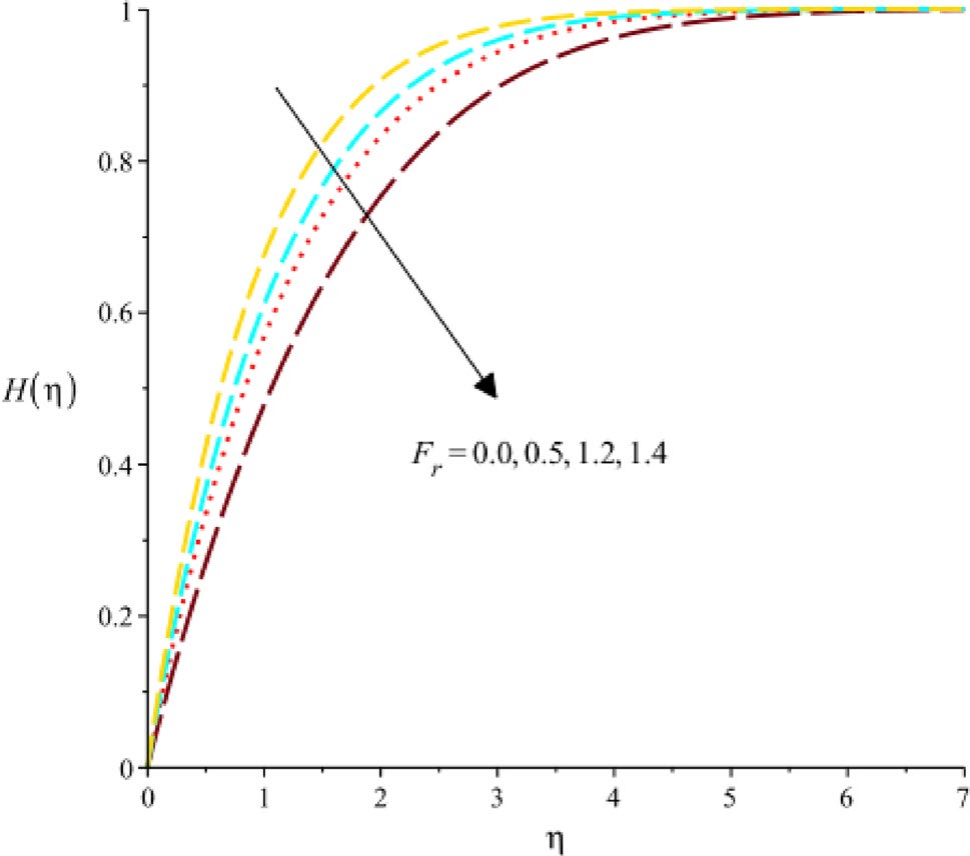
Variation of $H(\eta )$ against ${F}_{r}.$

**Figure 21:**
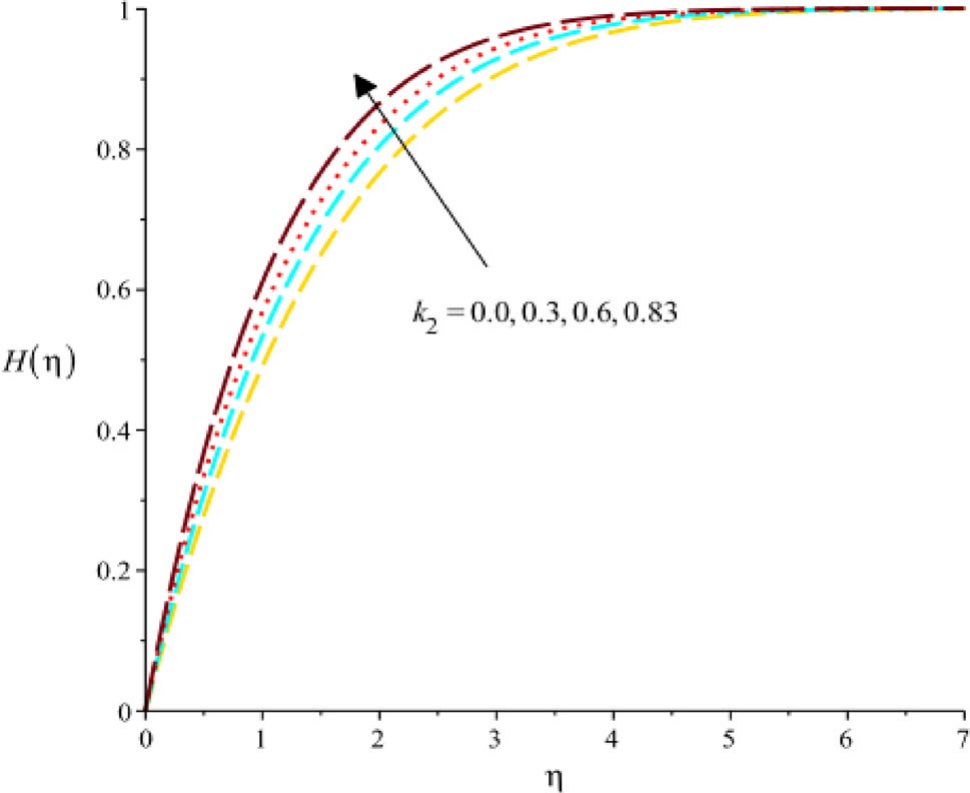
Variation of $H(\eta )$ against ${k}_{2}.$

Figure 18 exhibits the role of $Sc$ (Schmidt number) on concentration rate. It was evaluated that concentration rate enhances with increasing values of $Sc.$ The relative significance of momentum and mass transmission in a fluid is expressed by the dimensionless (Sc). It shows the proportion of mass diffusivity to momentum diffusivity (kinematic viscosity). It follows that when the Schmidt number rises, the momentum diffusivity is proportionally greater than the mass diffusivity. As the Schmidt number rises, the concentration rate, which is the rate at which the concentration of a species changes increases. A declination in concentration rate against change in ${k}_{1}$ (homogenous reaction) is experienced by Fig. 19. A rate equation that connects the rate of concentration change with regard to time is frequently used to explain the rate of a homogeneous chemical reaction. If a component affecting the rate constant in this equation is meant by the homogeneous reaction parameter, then an increase in the homogeneous reaction parameter would result in an increase in the rate constant. As a result, mathematically speaking, the concentration rate would rise along with the rate of change in concentration. The decreasing role of ${F}_{r}$ on concentrate rate curve is predicted by 20. It is based on the characterization fluid flow through porous medium is the Darcy’s parameter, also known as the Darcy’s permeability coefficient. It measures the porous medium’s permeability to fluid flow. An increase in Darcy’s parameter would result in a reduction in the concentration rate in the context of the concentration rate as presented in Fig. 20. The character of ${k}_{1}$ on concentration rate curve is observed by Fig. 21. Mathematical and physical explanations for a rise in the concentration rate when analyzing the impact of a heterogeneous response parameter on the concentration rate.

### Numerical study of divergent velocities and heat energy rate

**Figure 22:**
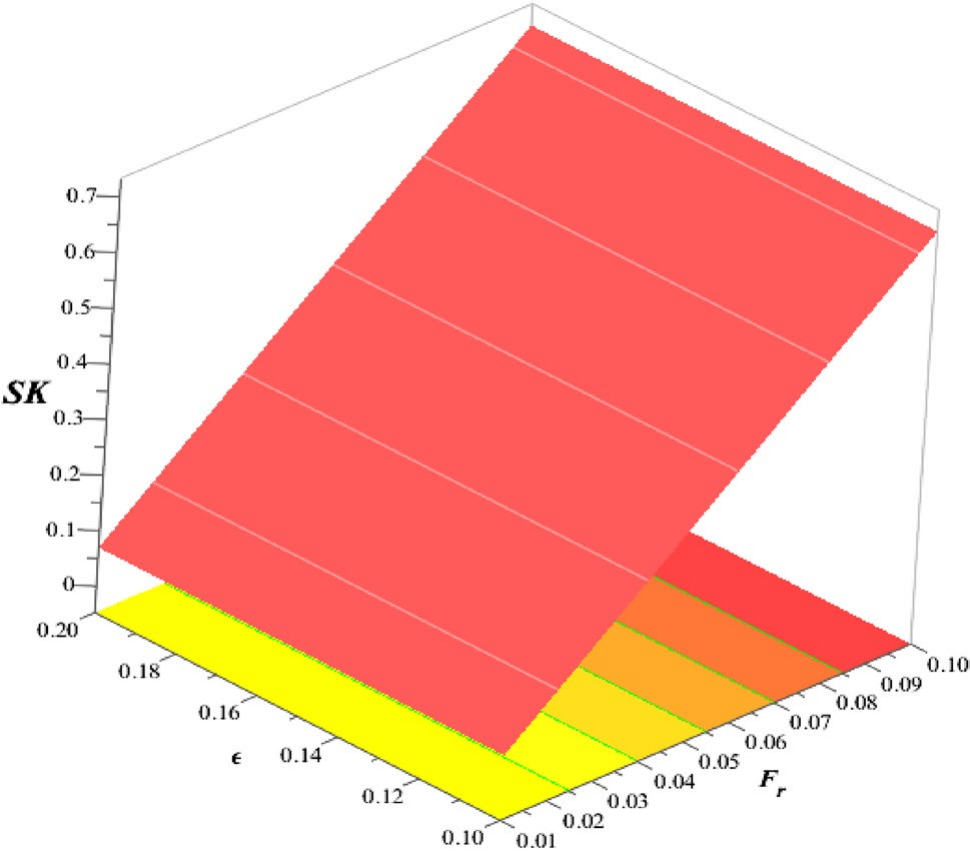
Variation of $\epsilon$ and ${F}_{r}$ on skin friction coefficient.

**Figure 23:**
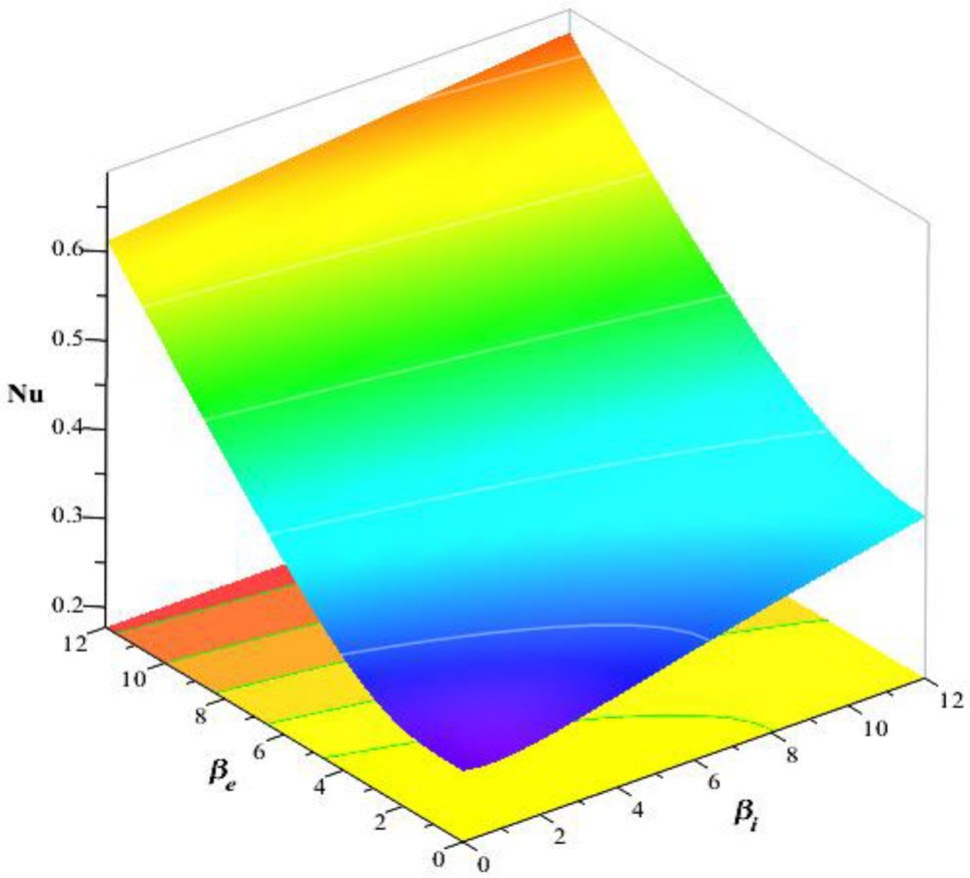
Variation of ${\beta }_{i}$ and ${\beta }_{e}$ on Nusselt number.

**Figure 24:**
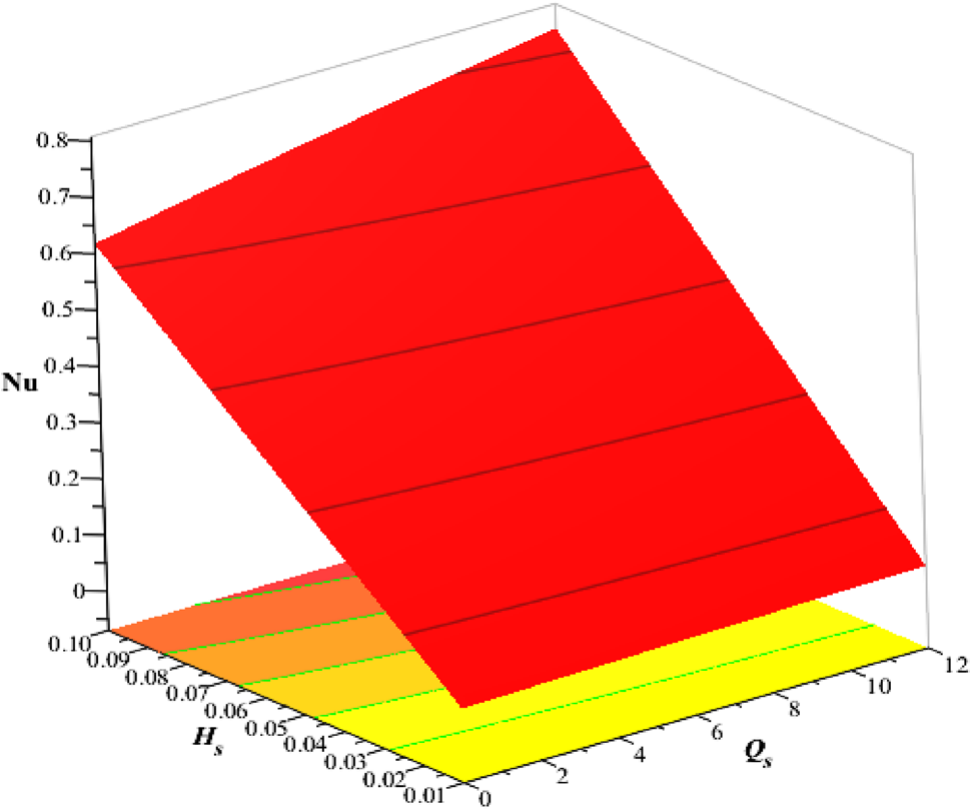
Variation of ${H}_{s}$ and ${Q}_{s}$ on Nusselt number.

Figure 22 estimates the observations of $\epsilon$ and ${F}_{r}$ on divergent velocity. It was evaluated that divergent velocity inclines when $\epsilon$ and ${F}_{r}$ are inclined. The frictional resistance between a fluid and a solid surface is represented by the skin friction coefficient, a dimensionless number. It is frequently used to evaluate the drag or resistance that a body encounters when traveling through a fluid. The skin friction coefficient increases with an increase in the Darcy’s number. Figures 23, 24 are plotted to experience variation of Nusselt number versus Hall number, heat source and ion slip parameters. It was determined that heat transfer rate increases with inclination of ${\beta }_{i}, {\beta }_{e}$ and heat source number. But reverse behavior is noted for ${H}_{s}$ on Nusselt number. Moreover, maximum transmission regrading heat energy has been observed for ternary hybrid nanofluid rather than nanofluid and hybrid nanofluid. Validity of results is shown in Table 3. Tables 4 and 5 reveal roles of $M, {\beta }_{e}, {H}_{s}$ and ${\beta }_{i}$ on wall shear stresses and temperature gradient considering $Si{O}_{2}$-$Ti{O}_{2}$/EG and $Si{O}_{2}$-$Ti{O}_{2}$-${Al}_{2}{O}_{3}$/EG. It was concluded that wall shear stresses are magnified versus change in $M$ but wall shear stresses are declined versus distribution in ${H}_{s}, {\beta }_{e}$ and ${\beta }_{i}.$ Moreover, rate of thermal production is declined when ${H}_{s}$ and $M$ are enhanced. Thermal rate increases versus change in ${\beta }_{e}, {H}_{s}$ and ${\beta }_{i}$. It was noticed that efficient improvement is investigated for $Si{O}_{2}$-$Ti{O}_{2}$-${Al}_{2}{O}_{3}$/EG rather than improvement of thermal rate for $Si{O}_{2}$-$Ti{O}_{2}$/EG.

**Table 3:** Validation results of temperature gradient and skin friction coefficients using finite element schema and homotropy analysis with published study^51^.

$n$	Homotropy analysis approach^51^			Finite element approach		
	${{\left(Re\right)}^{\frac{1}{n+1}}C}_{g}$	${{\left(Re\right)}^{\frac{1}{n+1}}C}_{f}$	$Nu{\left(Re\right)}^{\frac{-1}{n+1}}$	${{\left(Re\right)}^{\frac{1}{n+1}}C}_{g}$	${{\left(Re\right)}^{\frac{1}{n+1}}C}_{f}$	$Nu{\left(Re\right)}^{\frac{-1}{n+1}}$
1.0	2.69435000	2.19048887	3.40917100	2.690914904	2.190930414	3.4099398874
2.0	2.69506080	2.26914900	3.40830920	2.692049447	2.268473014	3.4080906490
3.0	2.71301081	2.36832100	3.74498558	2.717950993	2.737043018	3.7445043704

**Table 4:** Different values on wall shear stresses and temperature gradient involving $Si{O}_{2}$-$Ti{O}_{2}$/EG.

		$Si{O}_{2}$-$Ti{O}_{2}$/EG		
		$-{{\left(Re\right)}^{\frac{1}{n+1}}C}_{f}$	$-{{\left(Re\right)}^{\frac{1}{n+1}}C}_{f}$	$Nu{\left(Re\right)}^{\frac{-1}{n+1}}$
	0.0	0.2481577513	0.4377597443	1.371328218
$M$	0.53	0.2992583271	0.4702646017	1.272608561
	1.3	0.3362270769	0.4845789480	1.241056024
	0.0	0.2443460708	0.5730565674	0.6226188566
${\beta }_{e}$	0.3	0.2430984979	0.5612898031	0.3552415129
	0.6	0.2418698805	0.5500975106	0.3561245797
	0.0	0.2410975008	0.5432557971	0.2779640588
${\beta }_{i}$	0.56	0.2404411404	0.5375564297	0.2833083150
	1.45	0.2397898970	0.5320032911	0.2907157817
	− 1.8	0.2387155701	0.5230583680	0.05074841537
${H}_{s}$	0.6	0.2376546976	0.5144810431	0.04409620343
	1.5	0.2328373438	0.4784822389	0.03072529603
	0.0	0.2308664289	0.4650242440	0.5702344523
${Q}_{s}$	0.7	0.2288445112	0.4519064571	0.7034065817
	1.4	0.2278982474	0.4459928020	0.7625033837

**Table 5:** Different values on wall shear stresses and temperature gradient involving $Si{O}_{2}$-$Ti{O}_{2}$-${Al}_{2}{O}_{3}$/EG.

		$Si{O}_{2}$-$Ti{O}_{2}$-${Al}_{2}{O}_{3}$/EG		
		$-{{\left(Re\right)}^{\frac{1}{n+1}}C}_{f}$	$-{{\left(Re\right)}^{\frac{1}{n+1}}C}_{f}$	$Nu{\left(Re\right)}^{\frac{-1}{n+1}}$
	0.0	1.1904886710	1.694350208	2.40917113
$M$	0.53	1.1554243107	1.501664900	2.30651901
	1.3	1.1347482209	1.497378589	2.20772509
	0.0	1.2705803633	1.6942703140	2.43849881
${\beta }_{e}$	0.3	1.2640027687	1.6860945457	2.42020509
	0.6	1.2460154960	1.6778313403	2.40150551
	0.0	1.3371086729	1.4901776012	2.34830191
${\beta }_{i}$	0.56	1.3264654574	1.4663016003	2.37634399
	1.45	1.3151756349	1.4204056060	2.43062613
	− 1.8	1.3332425294	1.355977426	2.577700515
${H}_{s}$	0.6	1.3321736526	1.322343439	2.548361744
	1.5	1.3170275040	1.3084819103	2.411142785
	0.0	1.3560767583	1.9412607711	2.034334760
${Q}_{s}$	0.7	1.3453207320	1.9279511249	2.052359391
	1.4	1.3141940711	1.9087273411	2.078553134

## Conclusions

Dynamics of partially ions are analyzed in 3D power law model over stretching surface considering, solar thermal radiation, reactions based on heterogeneous and homogeneous. Ternary-hybrid nanomaterial is inserted in base fluid named as ethylene glycol. Thermal properties regarding nanoparticles are also inserted. Heat energy takes place under influence of heat source and Joule heating. Conclusions of present analysis are listed below.

- Thermal performance produced by $Si{O}_{2}$-$Ti{O}_{2}$-${Al}_{2}{O}_{3}$/EG is higher thermal performance produced by $Si{O}_{2}$-$Ti{O}_{2}$/EG.
- Ion slip and Hall forces are responsible for generating Joule heating mechanism that is responsible for reduction of velocity curve and generating shear stresses. Hence, tangential stresses are declined against increasing ${\beta }_{i}$ and ${\beta }_{e}.$
- It was included that motion for $Si{O}_{2}$-$Ti{O}_{2}$/EG is less than motion for $Si{O}_{2}$-$Ti{O}_{2}$-${Al}_{2}{O}_{3}$/EG. Further, motion declines when porosity parameter and Forchhiermer number are increased;
- Fluid temperature is enhanced by higher values of solar thermal radiation, heat source and Buoyancy parameters but Fluid temperature declines when ion slip number and Hall parameter are enhanced;
- Gradient of temperature (Nusselt number) enhances by enhancing values of solar thermal radiation, heat source and Buoyancy parameters. However, Gradient of temperature declines versus increasing values of ion slip number and Hall parameter;
- Opposite trends among heterogeneous (${k}_{2}$) and homogeneous (${k}_{1}$) reaction are investigated on concentration rate;
- Divergent velocity increased with increasing values of porosity parameter and Forchhiermer numbers.

## Data Availability

The data used to support this study are included in the Manuscript.

## List of symbols

$$W, V, U$$: Velocity components $$(m{s}^{-1})$$
$$z,x,y$$: Space coordinates ($$m$$)
$$T$$: Temperature ($$K$$)
$$G$$: Gravitational force ($$m{s}^{-2}$$)
$$\sigma$$: Electrical conductivity ($$S{m}^{-1}$$)
$${\beta }_{e}$$: Hall parameter
$${k}^{*}$$: Permeability number
$\beta$: Coefficient of thermal expansion (${K}^{-1}$)
${Q}_{0}$: Heat source number
${C}_{p}$: Specific heat capacitance
${k}_{2}$: Strength of heterogeneous
$$B$$: Heterogeneous reaction
$$c$$: Constant
${a}_{0}$: Reference homogeneous reaction
${T}_{f}$: Heat transfer coefficient ($K$)
${\mathrm{\rm A}}_{s}$: Coefficient of thermal radiation
$${F}_{r}$$: Forchheimer number
$${G}_{r}$$: Buoyancy parameter
$$Pr$$: Prandtl number
${H}_{s}$: Heat source number
$\delta$: Ratio of diffusion coefficient
$$\gamma$$: Biot number
${C}_{g}, {C}_{f}$: Skin friction coefficients
$Thnf$: Ternary hybrid nanofluid
ODEs: Ordinary differential equations
$n$: Power law index number
$$\rho$$: Density ($$Kg{m}^{-3}$$)
$${K}_{1}$$: Consistency coefficient
${B}_{0}$: Magnitude of magnetic field ($Ns{C}^{-1}{m}^{-1}$)
${\beta }_{i}$: Ion slip number
${F}_{st}$: Inertia coefficient number
$$\nu$$: Kinematic viscosity ($${m}^{2}{s}^{-1}$$)
$$k$$: Thermal conductivity ($$W{m}^{-1}$$)
$${A}_{sr}$$: Irradiance factor
$$A$$: Homogeneous reaction
$${k}_{c}$$: Chemical specie ($${s}^{-1}$$)
$${D}_{b}$$: Concentration ($$mol{m}^{-3}$$)
$${D}_{a}$$: Concentration ($$mol{m}^{-3}$$)
${h}_{f}$: Temperature of hot fluid
$${phi}_{1}, {phi}_{3}, {phi}_{2}$$: Volume fractions
$$\epsilon$$: Porosity parameter
$$M$$: Magnetic number
${\epsilon }_{1}$: Very small number
$${Q}_{sr}$$: Irradiance factor
$$Sc$$: Schmidt number
${k}_{1}$: Strength of homogenous
$$Re$$: Reynolds number
$$Nu$$: Nusselt number
$nf, bf$: Nanofluid and base fluid
$$e$$: Elements
