# Author Correction: Investigation of composed charged particles with suspension of ternary hybrid nanoparticles in 3D-power law model computed by Galerkin algorithm

**DOI:** 10.1038/s41598-023-47962-4

**Published:** 2023-11-24

**Authors:** Umar Nazir, Kanit Mukdasai, Muhammad Sohail, Abha Singh, Mohammed Theeb Alosaimi, Mashael Alanazi, Ayele Tulu

**Affiliations:** 1 Department of Mathematics, Faculty of Science, Khon Kaen University, Khon Kaen, 40002 Thailand; 2 Institute of Mathematics, Khwaja Fareed University of Engineering & Information Technology, Rahim Yar Khan, 64200 Pakistan; 3 Department of Basic Sciences, College of Sciences and Theoretical Studies, Dammam-branch, Saudi Electronic University, Riyadh, Saudi Arabia; 4 College of science and health professions, King Saud Bin Abdulaziz University for health Sciences, Riyadh, Saudi Arabia; 5 King Abdullah International Medical Research (KAIMRC), Riyadh, Saudi Arabia; 6 Ministry of National Guard-Health Affairs (MNGHA), Riyadh, Saudi Arabia; 7 Department of Mathematics, CNCS, Ambo University, Ambo, Ethiopia

Correction to: *Scientific Reports* 10.1038/s41598-023-41449-y, published online 12 September 2023

The original version of this Article omitted two Affiliations for Mohammed Theeb Alosaimi. The correct Affiliations are listed below.

1. College of science and health professions, King Saud Bin Abdulaziz University for Health Sciences, Riyadh, Saudi Arabia.

2. King Abdullah International Medical Research (KAIMRC), Riyadh, Saudi Arabia.

3. Ministry of National Guard-Health Affairs (MNGHA), Riyadh, Saudi Arabia.

The original Article has been corrected.

